# An improved ovine reference genome assembly to facilitate in-depth functional annotation of the sheep genome

**DOI:** 10.1093/gigascience/giab096

**Published:** 2022-02-04

**Authors:** Kimberly M Davenport, Derek M Bickhart, Kim Worley, Shwetha C Murali, Mazdak Salavati, Emily L Clark, Noelle E Cockett, Michael P Heaton, Timothy P L Smith, Brenda M Murdoch, Benjamin D Rosen

**Affiliations:** Department of Animal, Veterinary, and Food Sciences, University of Idaho, 875 Perimeter Dr, Moscow, ID 83843, USA; US Dairy Forage Research Center, USDA-ARS, 1925 Linden Drive, Madison, WI 53706, USA; Baylor College of Medicine, 1 Baylor Plaza, Houston, TX 77030, USA; Baylor College of Medicine, 1 Baylor Plaza, Houston, TX 77030, USA; The Roslin Institute, Royal (Dick) School of Veterinary Studies, The University of Edinburgh, Easter Bush Campus, Midlothian, EH25 9RG, UK; The Roslin Institute, Royal (Dick) School of Veterinary Studies, The University of Edinburgh, Easter Bush Campus, Midlothian, EH25 9RG, UK; Utah State University, Old Main Hill, Logan, UT 84322, USA; US Meat Animal Research Center, USDA-ARS, State Spur 18D, Clay Center, NE 68933, USA; US Meat Animal Research Center, USDA-ARS, State Spur 18D, Clay Center, NE 68933, USA; Department of Animal, Veterinary, and Food Sciences, University of Idaho, 875 Perimeter Dr, Moscow, ID 83843, USA; Animal Genomics and Improvement Laboratory, USDA-ARS, 10300 Baltimore Ave, Beltsville, MD 20705, USA

**Keywords:** Rambouillet, genome assembly, reference genome, sheep, Ovis aries

## Abstract

**Background:**

The domestic sheep (*Ovis aries*) is an important agricultural species raised for meat, wool, and milk across the world. A high-quality reference genome for this species enhances the ability to discover genetic mechanisms influencing biological traits. Furthermore, a high-quality reference genome allows for precise functional annotation of gene regulatory elements. The rapid advances in genome assembly algorithms and emergence of sequencing technologies with increasingly long reads provide the opportunity for an improved *de novo* assembly of the sheep reference genome.

**Findings:**

Short-read Illumina (55× coverage), long-read Pacific Biosciences (75× coverage), and Hi-C data from this ewe retrieved from public databases were combined with an additional 50× coverage of Oxford Nanopore data and assembled with canu v1.9. The assembled contigs were scaffolded using Hi-C data with Salsa v2.2, gaps filled with PBsuitev15.8.24, and polished with Nanopolish v0.12.5. After duplicate contig removal with PurgeDups v1.0.1, chromosomes were oriented and polished with 2 rounds of a pipeline that consisted of freebayes v1.3.1 to call variants, Merfin to validate them, and BCFtools to generate the consensus fasta. The ARS-UI_Ramb_v2.0 assembly is 2.63 Gb in length and has improved continuity (contig NG50 of 43.18 Mb), with a 19- and 38-fold decrease in the number of scaffolds compared with Oar_rambouillet_v1.0 and Oar_v4.0. ARS-UI_Ramb_v2.0 has greater per-base accuracy and fewer insertions and deletions identified from mapped RNA sequence than previous assemblies.

**Conclusions:**

The ARS-UI_Ramb_v2.0 assembly is a substantial improvement in contiguity that will optimize the functional annotation of the sheep genome and facilitate improved mapping accuracy of genetic variant and expression data for traits in sheep.

## Context

The domestic sheep (*Ovis aries*) is a globally important livestock species raised for a variety of purposes including meat, wool, and milk. Domestication likely occurred in multiple events ∼11,000 years ago [1–4]. Selection for desirable traits including meat, wool, and milk began ∼4,000–5,000 years ago [2, 4]. Modern sheep breeds exhibit a wide variety of phenotypes and adaptations to specific environments, e.g., the enhanced parasite tolerance evident in hair sheep [5, 6]. As many as 1,400 breeds of sheep exist today [7–9] including the Rambouillet breed developed in France from a Merino fine wool lineage, which is regarded for its ability to produce high-quality wool as well as meat products in production systems across the world [10, 11].

Genome research in sheep holds promise to improve efficiency and sustainability of production and reduce the environmental effects of animal agriculture [12]. The first sheep reference genome assembly was based on whole-genome shotgun (WGS) short-read sequencing, scaffolded by genetic linkage and radiation hybrid maps. The sequence came from 2 unrelated Texel breed sheep, with the first assembly draft (Oar_v3.1) (International Sheep Genomics Consortium, 2010) having a contig NG50, based on a 2.6 gigabase (Gb) genome size, of 39 kilobases kb and the update (Oar_v4.0) [13] boosting the NG50 metric to 145 kb. More recently, the Ovine Functional Annotation of Animal Genomes (FAANG) project proposed to perform a variety of genome annotation assays for dozens of tissues from a single animal [14, 15]. To maximize the success of assays that depend on mapping sequence data to a reference, the FAANG project assembled the genome of that animal, a female of the Rambouillet breed. The assembly, released in 2017 (Oar_rambouillet_v1.0, GenBank accession GCF_002742125; Worley et al., unpublished), is based on a combination of Pacific Biosciences RSII WGS long-read and Illumina short-read sequencing. It has an improved contig NG50 of 2.9 megabases (Mb) and is generally regarded as the official reference assembly for global sheep research.

The continued maturation of long-read sequencing technologies provided an opportunity to improve upon the sheep reference genome assembly. Because most of the proposed FAANG annotation assays had already been performed on the Rambouillet ewe, lung tissue from the same animal was chosen for DNA extraction. This allowed the use of existing long-read data to supplement new, longer-read, Oxford Nanopore PromethION sequencing. We report a *de novo* assembly of the same Rambouillet ewe used for Oar_rambouillet_v1.0, based on ∼50× coverage of nanopore reads (N50 47 kb) and 75× coverage Pacific Biosciences (PacBio) reads (N50 13 kb). The new assembly, ARS-UI_Ramb_v2.0, offers a 15-fold improvement in contiguity and increased accuracy, providing a basis for regulatory element annotation in the FAANG project and facilitating the discovery of biological mechanisms that influence traits important in global sheep research and production.

## Methods

### Sampling Strategy

**Figure 1:**
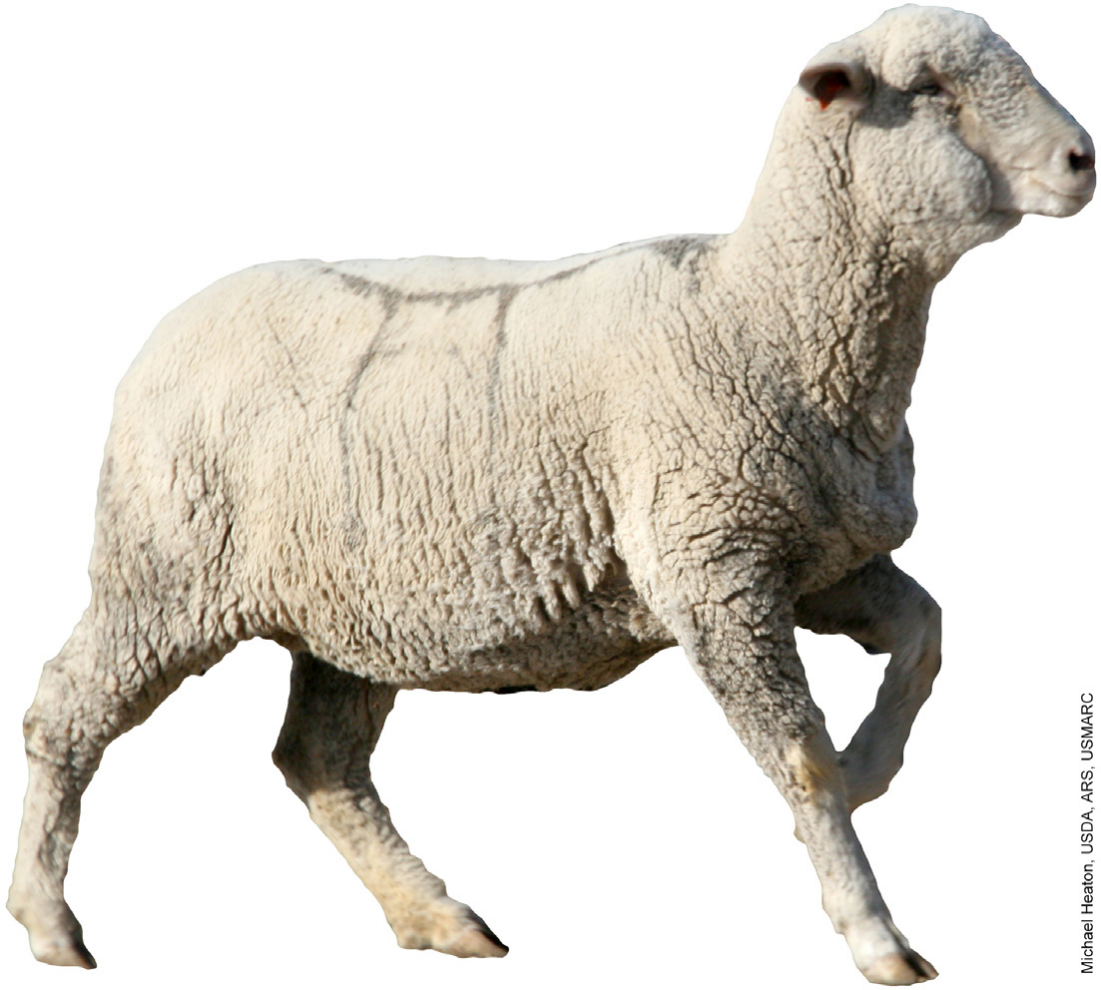
Image of Benz 2616 Rambouillet ewe selected for the ovine reference genome assembly.

The full-blood Rambouillet ewe used for this genome assembly (Benz 2616, USMARC ID 200,935,900) (Fig. 1) was selected by the FAANG project and acquired from the USDA. Tissues were collected post-mortem from the healthy 6-year-old ewe as approved by the Utah State University Institutional Animal Care and Use Committee. A full description of the tissue collection strategy is available in the FAANG Data Coordination Center [15, 16]. Details regarding the tissues collected from the animal are available under BioSample number SAMEG329607 [17].

### Sequencing and Data Acquisition

DNA was extracted from ∼50 mg of lung tissue using phenol:chloroform-based method as described [18]. Briefly, the frozen tissue was pulverized in a cryoPREP CP02 tissue disruption system (Covaris Inc., Woburn, MA, USA) as recommended by the manufacturer. The powdered tissue was transferred to a 50-mL conical tube and mixed in 200 µL of phosphate-buffered saline (Sigma-Aldrich, St. Louis, MO, USA). The tissue was then diluted in 10 mL of buffer TLB (100 mM sodium chloride, 10 mM Tris-HCl pH 8.0, 25 mM EDTA, 0.5% SDS) and mixed by vortexing, then incubated with 20 µL 10 mg/mL RNase A at 37°C for 1 hour with gentle shaking. Protein digestion was performed with 100 µL Proteinase K (20 mg/mL) at 50°C for 2 hours, with slow rotation of the tube to mix every 30 minutes. The lysate was distributed equally into a pair of 15 mL Phase Lock tubes (Quantabio, Beverly, MA, USA) and each tube received 5 mL of TE-saturated Phenol (Sigma-Aldrich, St. Louis, MO, USA) followed by mixing on a tube rotator at 20 RPM for 10 minutes at 22°C. The aqueous layer was collected after separating at 2300*g* for 10 minutes and transferred to another Phase Lock tube. A second extraction performed in the same way as the first was conducted using 2.5 mL phenol and 2.5 mL chloroform:isoamyl alcohol (Sigma). The final aqueous phase was transferred to a 50-mL conical tube and the DNA precipitated with 2 mL of 5 M ammonium acetate and 15 mL of ice-cold 100% ethanol. The DNA was pulled from the alcohol using a Pasteur pipet “hook” and placed in 10 mL of cold 70% ethanol to wash the pellet. The ethanol was poured off and the DNA pellet dried for 20–30 minutes, then dissolved in a dark drawer at room temperature for 48 hours in 1 mL of 10 mM Tris-Cl pH 8.5. Library preparation for Oxford Nanopore long-read sequencing was performed with an LSK-109 template preparation kit as recommended by the manufacturer (Oxford Nanopore, Oxford, UK) with modifications as described by Logsdon [18]. The ligated template was sequenced with a PromethION instrument using 4 R9.4 flow cells (Oxford Nanopore Technologies, Oxford, UK). Output as fast5 files were base-called with Guppy v3.1 [19]. Fastq files are available under the SRA accessions SRR17080040–3.

Sequence data used in the previous Oar_rambouillet_v1.0 assembly were retrieved from the SRA listed under project number PRJNA414087 [15]. PacBio RS II sequence generated from DNA extracted from whole blood was retrieved from SRX3445660, SRX3445661, SRX3445662, and SRX3445663. The Hi-C sequence data generated from liver using HindIII enzyme and sequenced at 150 bp paired end with an Illumina HiSeq X Ten were retrieved from SRX3399085 and SRX3399086. Short-read whole-genome sequencing from DNA extracted from whole blood collected from the Rambouillet ewe was performed with an Illumina HiSeq X Ten sequenced at 150 bp paired end and was retrieved from SRX3405602. Further details about these sequences can be found under the umbrella project number PRJNA414087. Short-read 45-bp paired-end whole-genome sequence from an Illumina Genome Analyzer II generated from Texel sheep used in previous genome assemblies was retrieved from the SRA under accessions SRX511533–65 (BioProject PRJNA169880).

### Assembly

Contigs were assembled with Oxford Nanopore and PacBio reads generated as described above using canu v1.8 (Canu, RRID:SCR_015880) through the trimmed reads stage of assembly. Parameters for contig construction were set as “batOptions = -dg 4 -db 4 -mo 1000” [20]. Canu v1.9 was used to complete the contig assembly because this update demonstrates better consensus generation of the overlapped contigs in the final step in the assembly process [21, 22]. The corrected error rate option was set as “correctedErrorRate = 0.105.”

### Scaffolding

Two Hi-C datasets from liver tissue from 2 different library preparations were retrieved as described above. The Hi-C reads were first aligned to the polished contigs using the Arima Genomics mapping pipeline [23]. This pipeline first maps paired-end reads individually with bwa-mem, then removes the 3′ end of reads identified as chimeric and span ligation junctions. Reads were then paired, filtered by mapping quality with samtools [24], and PCR duplicates removed with Picard [25]. The 2 Hi-C libraries were merged in the final step in the Arima pipeline to generate the merged BAM file. The BAM file was converted to a BED file for input into Salsa using the bedtools command bamToBed (BEDTools, RRID:SCR_006646) [26]. Salsa v2.2 was used for scaffolding by implementing “python run_pipeline.py -a contigs.fasta -l contigs.fasta.fai -b alignment.bed -e HindIII -o scaffolds -m yes” [27].

The Hi-C reads were aligned to the scaffolded assembly with the Arima Genomics mapping pipeline and then processed with PretextMap to visually evaluate the scaffolds as a contact map in PretextView [28]. The scaffolded assembly was also compared to Oar_rambouillet_v1.0 by aligning the 2 genomes with “minimap2 -cx asm5 Oar_rambouillet_v1.0_genomic.fasta scaffolds.fasta > alignment.paf” [29]. A dot plot of the alignment was visualized with D-Genies [30]. Scaffolds were edited on the basis of visual inspection of the contact map and dot plot, as well as the Hi-C alignment file. Scaffold joins and rearrangements were incorporated to the assembly using the agp2fasta mode of CombineFasta [31].

### Gap filling and polishing

Gap filling was completed with pbsuite v15.8.24 using both the PacBio and Oxford Nanopore reads. Nanopolish v0.12.5 (Nanopolish, RRID:SCR_016157) [32] with the NanoGrid parallel wrapper [33] was used with the raw fast5 files generated from the PromethION sequencing to polish the assembly. Duplicates were removed using PurgeDups v1.0.1 [34]. The chromosome orientation was confirmed in the polished assembly by identifying telomeres and centromeres using RepeatMasker v4.1.1 (RepeatMasker, RRID:SCR_012954) [35]. The mitochondrial genome was identified by aligning the previously annotated mitochondrial sequence from Oar_rambouillet_v1.0 (RefSeq NC_001941.1) to the assembly contigs and the start positions were matched. Chromosomes were oriented centromere to telomere and placed in chromosome number order. The final polishing with Illumina short-read data consisted of 2 rounds of freebayes v1.3.1 (FreeBayes, RRID:SCR_010761) [36] variant calling and BCFtools (SAMtools/BCFtools, RRID:SCR_005227) [24] consensus. Variants used for polishing with both Nanopolish and freebayes/BCFtools were screened with Merfin [37], which evaluates the *k*-mer consequences of variant calls and filters unsupported variants.

### RNA sequencing

RNA sequencing data were generated from 5 tissues including skin, thalamus, pituitary, lymph node (mesenteric), and abomasum pylorus collected from the animal used to assemble the reference genome. Details regarding the RNA isolation protocol, library preparation, and sequencing as well as the raw data can be found in GenBank under BioProject PRJEB35292, specifically under SRA run numbers ERR3665717 (skin), ERR3728435 (thalamus), ERR3650379 (pituitary), ERR3665711 (lymph node mesenteric), and ERR3650373 (abomasum pylorus). Reads were trimmed with Trim Galore v0.6.4 [38] and alignment to both Rambouillet genomes was performed with STAR v2.7 using default parameters [39]. Indels were identified with bcftools mpileup, filtering allele depth (AD) at > 5 [40].

### Annotation

The annotation for ARS-UI_Ramb_v2.0, NCBI Ovis aries Annotation Release 104, is available in RefSeq and other NCBI genome resources [41].

Here we also provide a liftover of the annotation for Oar_rambouillet_v1.0 onto ARS-UI_Ramb_v2.0. The annotation used for the liftover was NCBI v103 GCF_002742125.1_Oar_rambouillet_v1.0_genomic.fna.gz. The GFF3 format gene annotation file was prepared for processing using liftOff v1.5.2 [42]. A set of matching chromosome names for Oar_rambouillet_v1.0 and ARS-UI_Ramb_v2.0 were generated according to the instructions for liftOff (paste -d “,” <(cut -d' ‘ -f1 ramb1.chr) <(cut -d’ ' -f1 ramb2.chr) > chroms.txt). The GFF file (annotation Ramb1LO2) generated by liftOff is included in Supplementary File 1 (Ramb_v1.0_NCBI103_lifted_over_ARS-UI_Ramb_v2.0.gff.gz).

To compare the breakdown of transcripts captured by the 3 annotations (Oar_Rambouillet_v1.0, Ramb1LO2 [liftover], and ARS-UI_Ramb_v2.0), we generated transcript expression estimates using Kallisto v0.44.0 (kallisto, RRID:SCR_016582) [43]. For the lifted over gene annotation the GFF file (Ramb_v1.0_NCBI103_lifted_over_ARS-UI_Ramb_v2.0.gff.gz) was used to generate transcriptome sequence FASTA files, as a Kallisto index, for transcript expression estimation. Briefly, exonic blocks were extracted from the GFF3 file using the awk command (awk “($3∼/exon/)” input.gff). The getfasta and groupby plugins from bedtools v2.30.0 [44] were used to extract the exonic sequences and group them by transcript name. Exonic sequences for each transcript were appended in the correct order, to produce the complete sequence for each transcript. The FASTA format file for the whole transcriptome was created using all of the transcript-level FASTA sequences for the liftover annotation Ramb1LO2 (Supplementary File 2; Ramb1LO2_NCBI103_geneBank_rna.fa). The set of scripts used for this step are included in Supplementary File 3. The Kallisto indices for Oar_Rambouillet_v1.0 (GCF_002742125.1_Oar_rambouillet_v1.0_rna.fna.gz), Ramb1LO2 (liftover; Ramb1LO2_NCBI103_geneBank_rna.fa), and ARS-UI_Ramb_v2.0 (GCF_016772045.1_ARS-UI_Ramb_v2.0_rna.fna.gz) were then used with the RNA-Seq data from the 61 tissues from Benz2616 (GenBank BioProject PRJNA414087 and PRJEB35292) to estimate transcript-level expression for every tissue as transcript per million mapped reads (TPM) and compared across the 3 annotations.

## Data Validation and Quality Control

### Assembly Quality Statistics

**Figure 2:**
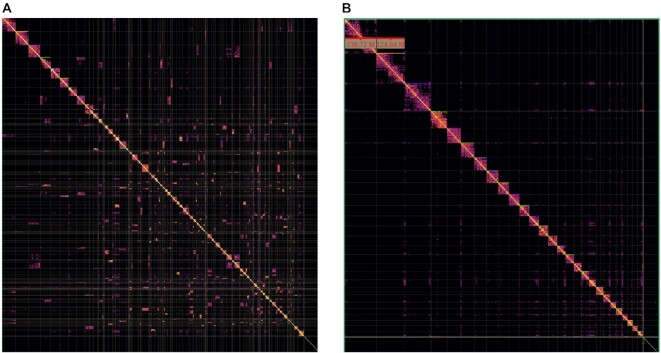
Hi-C contact map comparison of ARS-UI_Ramb_v2.0 (A) directly after scaffolding and before manual curation and (B) after manual curation with scaffold rearrangements and joins.

The 4 flow cells of PromethION data produced 136 Gb of WGS sequence (∼51× coverage) in reads having a read N50 of 47 kb. The initial generation of contigs used this data as well as 198.1 Gb of RSII data with a read N50 of 12.9 kb. The ARS-UI_Ramb_v2.0 assembly was submitted to NCBI GenBank under accession number GCF_016772045.1, and statistics of contigs and scaffolds following initial polishing, scaffolding with Hi-C data, and manual editing, gap-filling, and final polishing, are shown in Table 1. The assembly improved on the Oar_v4.0/Oar_rambouillet_v1.0 sheep reference assemblies in all continuity measures (Table 1) including a 286/17-fold increase in contig N50 (the size of the shortest contig for which all larger contigs contain half of the total assembly), a 214/33-fold reduction in the number of contigs in the assembly and concomitant 209/13-fold reduction of contig L50 (the number of contigs making up half of the total assembly), and 38/19-fold reduction in total number of scaffolds. Manual curation of scaffolds using Hi-C data improved scaffold continuity and led to chromosome-length scaffolds (Fig. 2).

**Figure 5:**
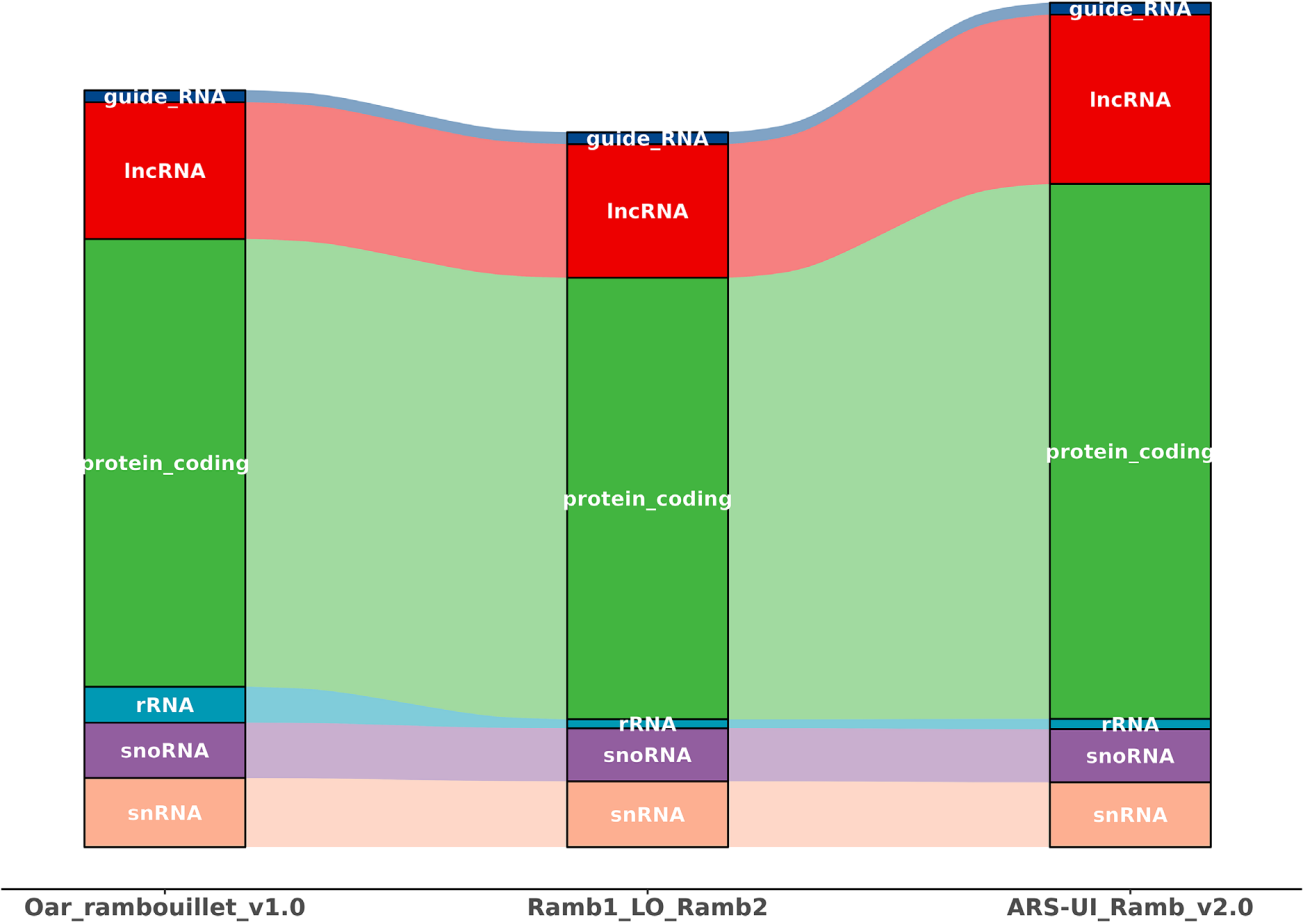
Kallisto comparison of the number of expressed transcripts for the RNA-Seq dataset of 61 tissue samples from Benz2616, across the 3 annotations (Oar_Rambouillet_v1.0, Ramb1LO2 [liftover], and ARS-UI_Ramb_v2.0). lncRNA: long non-coding RNA; rRNA: ribosomal RNA; snoRNA: small nucleolar RNA; snRNA: small nuclear RNA.

**Table 1:** Assembly: quality statistics comparison

Assembly statistic	ARS-UI_Ramb_v2.0	Oar_rambouillet_v1.0	Oar_v4.0	Description
Total Length (Mb)	2,628.15	2,869.91	2,615.52	Assembly length in Mb
Contig No.	226	7,486	48,482	Total number of contigs
Contig NG50 (bp)	43,178,051	2,850,956	145,655	Half the length of the genome is in contigs of this size or greater, based on a 2,600 Mb genome
Contig LG50 (No. of contigs)	24	263	5,206	The smallest number of contigs whose length sum make up half of the genome size
Scaffold No.	142	2,641	5,466	Total number of scaffolds and unplaced contigs in the assembly
merQV	44.7721*	32.1705*	31.9131**	*k*-mer–based quality from Merqury, which estimates the frequency of consensus errors in the assembly [48]
merErrorRate	0.000033327*	0.00060662*	0.000643714**	*k*-mer–based error rate from Merqury, which estimates error rate of the assembly based on errors in *k*-mers [48]
merCompleteness	93.0479*	93.4711*	92.2182**	Proportion of complete assembly estimated by Merqury based on “reliable” *k*-mers, or *k*-mers unlikely to be caused by sequencing error [48]
baseQV	41.84*	40.69*	32.40**	SNP and INDEL quality value estimated from short-read data mapped to the assembly [49]
Unmap%	0.96*	1.00*	0.73**	Percentage of short reads that are unmapped to each assembly [49]
COMPLETESC	93.9	93.0	91.2	Percent of complete, single-copy BUSCOs
COMPLETEDUP	2.1	2.6	1.6	Percent of complete, duplicated BUSCOs
FRAGMENT	0.9	1.1	2.4	Percent of fragmented BUSCOs
MISSING	3.1	3.3	4.8	Percent of missing BUSCOs

* Short-read sequencing from the Rambouillet ewe used to assemble both ARS-UI_Ramb_v2.0 and Oar_rambouillet_v1.0 was used in these quality values.

** Short-read sequencing from the Texel animal used to assemble Oar_v4.0 was used in these quality values.

The Themis-ASM pipeline [45] was implemented to further assess assembly quality and compare sheep genome assemblies. Short-read sequence from both the Rambouillet ewe used in this assembly and Texel sheep from previous sheep genome assemblies were used to compare ARS-UI_Ramb_v2.0 with Oar_rambouillet_v1.0 and Oar_v4.0 assemblies.

The *k*-mer–based quality value and error rates improved with ARS-UI_Ramb_v2.0 compared with Oar_rambouillet_v1.0 and Oar_v4.0. This is also reflected in the proportion of complete assembly based on *k*-mers (merCompleteness), which is similar between ARS-UI_Ramb_v2.0 and Oar_rambouillet_v1.0 and both are higher than Oar_v4.0. Furthermore, the single-nucletide polymorphism (SNP) and indel quality value (baseQV) were greatest overall in ARS-UI_Ramb_v2.0 (41.84), followed by Oar_rambouillet_v1.0 (40.69) and Oar_v4.0 (32.40). The percentage of short reads not mapped to the genome was ≤1% in all 3 assemblies.

The completeness of ARS-UI_Ramb_v2.0 was evaluated by examining the presence or absence of evolutionarily conserved genes in each assembly using BUSCO (BUSCO, RRID:SCR_015008) v5.2.2 scores with the cetartiodactyla_odb10 dataset and metaeuk gene predictor [46]. The percent of single-copy complete BUSCOs was higher (93.9%) in ARS-UI_Ramb_v2.0 when compared with Oar_rambouillet_v1.0 (93.0%) and Oar_v4.0 (91.2%). Complete duplicated BUSCO percentage was highest in Oar_rambouillet_v1.0 (2.6%) compared with ARS-UI_Ramb_v2.0 (2.1%), and lowest in Oar_v4.0 (1.6%). Furthermore, ARS-UI_Ramb_v2.0 had the lowest percent of fragmented and missing BUSCOs (0.9% and 3.1%, respectively) compared with Oar_rambouillet_v1.0 (1.1% and 3.3%, respectively) and Oar_v4.0 (2.4% and 4.8%, respectively).

**Figure 3:**
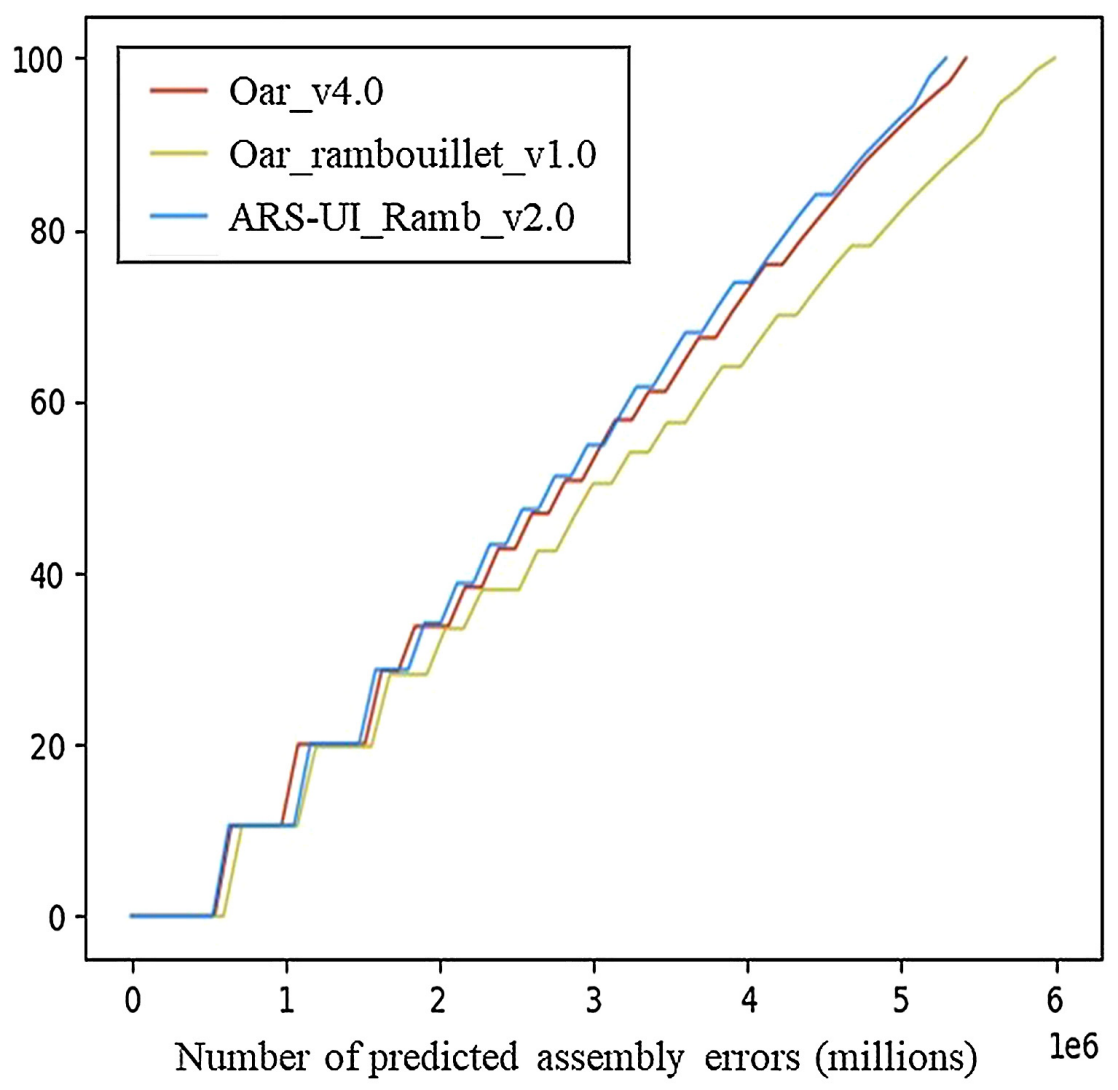
Assembly error comparison between ARS-UI_Ramb_v2.0, Oar_rambouillet_v1.0, and Oar_v4.0 in a feature response curve displaying sorted lengths of the assemblies with the fewest errors.

The 3 sheep genome assemblies were also compared with a feature response curve in which the quality of the assembly is analyzed as a function of the features, or maximum number of possible errors, allowed in the contigs (Fig. 3) [47]. Both the ARS-UI_Ramb_v2.0 and Oar_v4.0 feature response curves peak higher and to the left of Oar_rambouillet_v1.0, which indicates fewer errors in these assemblies (Fig. 3). The ARS-UI_Ramb_v2.0 genome also has fewer regions with either low or high coverage overall and for paired reads, suggesting fewer coverage issues, as well as fewer improperly paired or unmapped single reads when compared with other assemblies (Table 2). The number of high Comp/Expansion (CE) statistics in ARS-UI_Ramb_v2.0 was intermediate between Oar_rambouillet_v1.0 (higher) and Oar_v4.0 (lower); however, this latest assembly had the lowest number of regions with low CE statistics.

**Table 2:** : Specific feature counts for each genome and descriptions.

Features	ARS-UI_Ramb_v2.0	Oar_rambouillet_v1.0	Oar_v4.0	Description
LOW_COV_PE	7212	95166	89103	Low read coverage areas
LOW_NORM_COV_PE	2990	24381	26860	Low coverage of normal paired end reads
HIGH_SPAN_PE	6522	22628	33232	Regions with high numbers of inter-contig paired end reads
HIGH_COV_PE	2051	3630	26276	Regions with high read coverage
HIGH_NORM_COV_PE	2366	2633	1875	Regions with high coverage of normal paired end reads
HIGH_OUTIE_PE	2514	28766	37495	Regions with high counts of improperly paired reads
HIGH_SINGLE_PE	0	0	0	Regions with high counts of single unmapped reads
STRECH_PE	74	84	67	Regions with high Comp/Expansion (CE) statistics
COMPR_PE	87	92	44	Regions with low Comp/Expansion (CE) statistics

**Figure 4:**
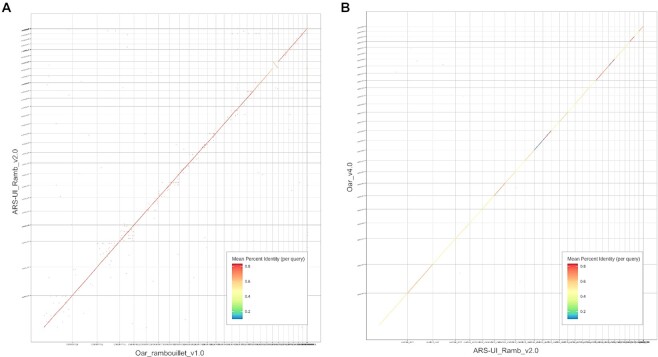
Dot plot comparison of genome assemblies between (A) ARS-UI_Ramb_v2.0 and Oar_rambouillet_v1.0, and (B) ARS-UI_Ramb_v2.0 and Oar_v4.0.

Comparative alignment of ARS-UI_Ramb_v2.0 with previous assemblies Oar_rambouillet_v1.0 and Oar_v4.0 and visualization with a dot plot revealed a high amount of agreement between assemblies (Fig. 4). Interestingly, chromosome 11 was improperly oriented in Oar_rambouillet_v1.0, and after confirming centromere and telomere locations on this chromosome, this was resolved in the ARS-UI_Ramb_v2.0 assembly. The percent identity between ARS-UI_Ramb_v2.0 is very high when compared with Oar_rambouillet_v1.0, which was expected considering that the same animal was used in both assemblies. However, Oar_v4.0 was assembled from Texel sheep, which is apparent in the percent identity in the dot plot.

In summary, ARS-UI_Ramb_v2.0 offers greater contiguity, improved quality, more complete BUSCOs, and fewer assembly errors when compared with previous assemblies.

### RNA sequencing alignment

Insertions and deletions (indels) in the ARS-UI_Ramb_v2.0 assembly were characterized and compared with Oar_rambouillet_v1.0 by mapping 150 bp paired-end RNA-seq data from skin, thalamus, pituitary, lymph node (mesenteric), and abomasum pylorus generated from the same animal used to assemble the reference genome (Table 3). In all 5 tissues, ARS-UI_Ramb_v2.0 had nearly half of the number of indels compared with Oar_rambouillet_v1.0. Most indels identified in both assemblies were 1 bp in length. The ARS-UI_Ramb_v2.0 had a greater number of uniquely mapped reads in each tissue when compared with Oar_rambouillet_v1.0, leading to an approximate 2% increase in the percent of uniquely mapped reads in most tissues except pituitary, which saw 13% improvement. The number of reads that mapped to multiple loci decreased in the new assembly by 12.59% in pituitary, and 1–2% in other tissues. Furthermore, ARS-UI_Ramb_v2.0 had fewer unmapped reads than Oar_rambouillet_v1.0 across all 5 tissues by an average of 0.15%.

**Table 3:** RNA-seq: alignment statistics to ARS-UI_Ramb_v2.0 and Oar_rambouillet_v1.0 from 5 different tissues

Tissue	Genome*	No. input reads	Reads uniquely mapped		Reads multi-mapped		Reads unmapped		No. indels
			No.	%	No.	%	No.	%	
Skin	v2.0	62,630,134	53,990,480	86.20	6,684,213	10.67	1,955,441	3.12	962
	v1.0		52,523,732	83.86	8,114,599	12.96	1,991,803	3.18	2,512
	Δ	N/A	1,466,748	2.34	−1,430,386	−2.29	−36,362	−0.06	−1,550
Thalamus	v2.0	54,655,873	45,721,452	83.65	5,414,620	9.91	3,519,801	6.44	649
	v1.0		44,904,096	82.16	6,126,363	11.21	3,625,414	6.63	1,054
	Δ	N/A	817,356	1.49	−711,743	−1.30	−105,613	−0.19	−405
Pituitary	v2.0	43,368,663	39,710,031	91.56	2,405,103	5.55	1,253,529	2.89	604
	v1.0		34,115,417	78.66	7,866,251	18.14	1,386,995	3.20	960
	Δ	N/A	5,594,614	12.90	−5,461,148	−12.59	−133,466	-0.31	−356
Lymph node—	v2.0	43,673,576	38,819,419	88.88	3,562,121	8.16	1,292,036	2.96	684
mesenteric	v1.0		38,296,065	87.69	4,057,915	9.29	1,319,596	3.02	999
	Δ	N/A	523,354	1.19	−495,794	−1.13	−27,560	−0.06	−315
Abomasum	v2.0	45,977,534	41,018,529	89.21	2,978,042	6.48	1,980,963	4.31	512
pylorus	v1.0		40,403,981	87.88	3,533,015	7.68	2,040,538	4.44	846
	Δ	N/A	614,548	1.33	−554,973	−1.20	−59,575	−0.13	−334

* Genomes include v2.0 (ARS-UI_Ramb_v2.0) and v1.0 (Oar_rambouillet_v1.0) and the difference (Δ).

### Annotation

The ARS-UI_Ramb_v2.0 annotation represents a substantial improvement over the annotation on Oar_rambouillet_v1.0. For example, for ARS-UI_Ramb_v2.0 16,500 coding genes have an ortholog to human (compared to 16,319 for Oar_rambouillet_v1.0), and the BUSCO scores demonstrate that 99.1% of the gene models (cetartiodactyla_odb10) are complete in the new annotation versus 98.8% in the previous one. The annotation for ARS-UI_Ramb_v2.0 includes Iso-Sequencing for 8 tissues to improve contiguity of gene models, and CAGE sequencing for 56 tissues to define transcription start sites, that were not used to annotate Oar_rambouillet_v1.0.

Using Kallisto we compared the number of expressed transcripts, for the RNA-Seq dataset of 61 tissue samples from Benz2616, across the 3 annotations (Oar_Rambouillet_v1.0, Ramb1LO2 [liftover], and ARS-UI_Ramb_v2.0). There was a considerable increase in the number of transcripts captured by the annotation for ARS-UI_Ramb_v2.0 (60,064) relative to Oar_Rambouillet_v1.0 (42,058) and the liftover annotation (Ramb1LO2) (40,910) (Table 4 and Fig. 5). This equates to ∼20,000 new annotated gene models for ARS-UI_Ramb_v2.0 and further reflects the substantial improvement over the annotation for Oar_Rambouillet_v1.0.

**Table 4:** : Expressed transcripts (TPM > 0) in Benz2616 tissues (*n* = 61) based on Oar_rambouillet_v1.0 and ARS-UI_Ramb_v2.0 and lift over (LO) (RefSeq v103 & 104, respectively).

Gene Biotype	Oar_rambouillet_v1.0	Oar_rambouillet_v1.0 LO	ARS-UI_Ramb_v2.0	LO vs. Oar_rambouillet_v1.0	LO vs. ARS-UI_Ramb_v2.0	Oar_rambouillet_v1.0 vs. ARS-UI_Ramb_v2.0
Guide RNA	30	29	30	–1	–1	0
lncRNA	3929	3752	6018	–177	–2266	–2089
Protein coding	42058	40910	60064	–1148	–19154	–18006
rRNA	272	17	22	–255	–5	250
snoRNA	644	590	593	–54	–3	51
snRNA	997	907	879	–90	28	118

The lifted over annotation that we have generated will provide a resource for those who wish to compare their results for ARS-UI_Ramb_v2.0 to previous work using Oar_Rambouillet_v1.0.  Only 2.7% of protein-coding transcripts were lost (1,148) lifting over the annotation for Oar_Rambouillet_v1.0 onto ARS-UI_Ramb_v2.0. According to the annotation report provided by NCBI [51], 70% of the annotations were identical or had only minor changes between and Oar_Rambouillet_v1.0 and ARS-UI_Ramb_v2.0.

### Reuse potential

The ARS-UI_Ramb_v2.0 genome assembly serves as a reference for genetic investigation of traits important in sheep research and production across the world. This genome is assembled from the same animal used in the Ovine FAANG Project, which provides a high-quality basis for epigenetic annotation to serve the international sheep genomics community and scientific community at large.

## Data Availability

The datasets supporting the results of this article are available in the RefSeq repository, GCF_016772045.1, and in the GigaScience Database [50]. RNA sequencing data are available under BioProject PRJEB35292. The full report for the annotation release is available at https://www.ncbi.nlm.nih.gov/genome/annotation_euk/Ovis_aries/104/. Ovis aries Annotation Release 104 is also available in RefSeq and other NCBI genome resources [41].

## Additional Files

**Supplementary File 1**. Annotation lifted over from Oar_rambouillet_v1.0 to ARS-UI_Ramb_v2.0.

**Supplementary File 2**. RNA file from annotation lift over.

**Supplementary File 3**. Scripts from annotation lift over.

giab096_GIGA-D-21-00165_Original_Submission

giab096_GIGA-D-21-00165_Revision_1

giab096_GIGA-D-21-00165_Revision_2

giab096_Response_to_Reviewer_Comments_Revision_1

giab096_Reviewer_1_Report_Original_Submission Aaron Shafer -- 8/30/2021 Reviewed

giab096_Reviewer_2_Report_Original_Submission Elizabeth Ross -- 10/10/2021 Reviewed

giab096_Supplemental_Files

## Abbreviations

bp: base pairs; BUSCO: Benchmarking Universal Single-Copy Orthologs; CAGE: cap analysis gene expression; EDTA: ethylenediaminetetraacetic acid; FAANG: Ovine Functional Annotation of Animal Genomes; Gb: gigabase pairs; kb: kilobase pairs; Mb: megabase pairs; NCBI: National Center for Biotechnology Information; PacBio: Pacific Biosciences; SDS: sodium dodecyl sulfate; SNP: single-nucletide polymorphism; USDA: United States Department of Agriculture; WGS: whole-genome shotgun.

## Funding

Funding was provided by Agriculture and Food Research Initiative Competitive grants from the USDA National Institute of Food and Agriculture supporting improvements of the sheep genomes (2013–67015-21228) and FAANG activities (2013–67015-21372, 2017–67016-26301). Additional funding was received from the International Sheep Genome Consortium (217201191442) and infrastructure support from a grant to R. Gibbs from the NIH NHGRI Large-Scale Sequencing Program (U54 HG003273).

D.M.B. was supported by appropriated USDA CRIS project 5090–31000-026–00-D. T.P.L.S. was supported by appropriated USDA CRIS Project 3040–31000-100–00D. B.D.R. was supported by appropriated USDA CRIS Project 8042–31000-001–00-D. The USDA does not endorse any products or services. Mentioning of trade names is for information purposes only. The USDA is an equal opportunity employer.

## Authors' Contributions

B.M.M., T.P.L.S., D.M.B., and B.D.R. conceptualized the study. B.M.M., N.E.C., M.P.H., and T.P.L.S. selected the animal and collected samples. K.W. and S.C.M. facilitated the generation of RSII, short-read, and Hi-C data. T.P.L.S. facilitated the nanopore long-read data generation. K.M.D., D.M.B., T.P.L.S., B.M.M., and B.D.R. performed the genome assembly, scaffolding, RNA-sequencing alignment, polishing, and quality control. M.S. and E.L.C. contributed the section describing the LiftOff annotation and comparative analysis of transcript expression estimates for the 3 annotations. K.M.D., D.M.B., T.P.L.S., B.M.M., and B.D.R. generated tables and figures and drafted the manuscript. K.M.D., D.M.B., K.W., S.C.M., N.E.C., T.P.L.S., B.M.M., and B.D.R. edited the manuscript. All authors contributed to the article and approved the final version.

## Conflict of Interest

The authors have no conflicts of interest.
