# Supplementary material for: An improved ovine reference genome assembly to facilitate in-depth functional annotation of the sheep genome
Source: Gigascience. 2022 Feb 4;11:giab096. doi: 10.1093/gigascience/giab096 (PMC8848310; doi:10.1093/gigascience/giab096)

GigaScience

An improved ovine reference genome assembly to facilitate in depth functional annotation of the sheep genome

--Manuscript Draft--

**Manuscript Number:**	GIGA-D-21-00165R1
**Full Title:**	An improved ovine reference genome assembly to facilitate in depth functional annotation of the sheep genome
**Article Type:**	Data Note

Funding Information:

Funder	Recipient
National Institute of Food and Agriculture (2013-67015-21228)	Dr. Kim C. Worley
National Institute of Food and Agriculture (2013-67015-21372)	Dr. Noelle E. Cockett
National Institute of Food and Agriculture (2017-67016-26301)	Dr. Brenda M. Murdoch
International Sheep Genomics Consortium (217201191442)	Dr. Kim C. Worley
Agricultural Research Service (5090-31000-026-00-D)	Dr. Derek M. Bickhart
Agricultural Research Service (3040-31000-100-00D)	Dr. Timothy P.L. Smith
Agricultural Research Service (8042-31000-001-00-D)	Dr. Benjamin D. Rosen

Abstract:

Background

The domestic sheep (Ovis aries) is an important agricultural species raised for meat, wool, and milk across the world. A high-quality reference genome for this species enhances the ability to discover genetic mechanisms influencing biological traits. Further, a high-quality reference genome allows for precise functional annotation of gene regulatory elements. The rapid advances in genome assembly algorithms and emergence of sequencing technologies with increasingly long reads provide the opportunity for an improved de novo assembly of the sheep reference genome.

Findings

Short-read Illumina (55x coverage), long-read PacBio (75x coverage), and Hi-C data from this ewe retrieved from public databases were combined with an additional 50x coverage of Oxford Nanopore data and assembled with canu v1.9. The assembled contigs were scaffolded using Hi-C data with Salsa v2.2, gaps filled with PBsuitev15.8.24, and polished with Nanopolish v0.12.5. After duplicate contig removal with PurgeDups v1.0.1, chromosomes were oriented and polished with two rounds of a pipeline which consisted of freebayes v1.3.1 to call variants, Merfin to validate them, and BCFtools to generate the consensus fasta. The ARS-UI_Ramb_v2.0 assembly is 2.63 Gb in length and has improved continuity (contig NG50 of 43.18 Mb) with a 19-fold and 38-fold decrease in the number of scaffolds compared with Oar_rambouillet_v1.0 and Oar_v4.0. ARS-UI_Ramb_v2.0 has greater per-base accuracy and fewer insertions and deletions identified from mapped RNA sequence than previous assemblies.

Conclusions

The ARS-UI_Ramb_v2.0 assembly is a substantial improvement in contiguity that will optimize the functional annotation of the sheep genome and facilitate improved mapping accuracy of genetic variant and expression data for traits in sheep.

Corresponding Author: Benjamin D Rosen

UNITED STATES

Corresponding Author Secondary

Powered by Editorial Manager® and ProduXion Manager® from Aries Systems Corporation

Information:	
Corresponding Author's Institution:	
Corresponding Author's Secondary Institution:	
First Author:	Kimberly M Davenport, M.S.
First Author Secondary Information:	
Order of Authors:	Kimberly M Davenport, M.S.
	Derek M. Bickhart
	Kim C. Worley
	Shwetha C. Murali
	Mazdak Salavati
	Emily L. Clark
	Noelle E. Cockett
	Michael P. Heaton
	Timothy P.L. Smith
	Brenda M. Murdoch
	Benjamin D. Rosen
Order of Authors Secondary Information:	
Response to Reviewers:	Response to Reviewer Comments<br><br>Reviewer #1: My comments are minimal as the paper is succint. The authors present an improved genome, largely in the form of contiguity, and provide a number of statistics to support their argument. There are literally dozens upon dozens of different ways to assemble and polish a genome and I see no value in suggesting changes in this regard as the approach more-or-less reflects the state-of-the-art.<br>Response: Thank you to Reviewer 1 for the thorough review of this manuscript and helpful suggestions for improvement.<br><br>I also might question the description of "substantial improvement" as this really reflects the improvement in contiguity and less so the BUSCO, annotation.<br>Response: The sentence in Line 76 was revised to specify the improvement in contiguity of the new genome. The sentence now reads, “The ARS-UI_Ramb_v2.0 assembly is a substantial improvement in contiguity that will optimize the functional annotation of the sheep genome and facilitate improved mapping accuracy of genetic variant and expression data for traits in sheep.”<br><br>Also, scaffold L50 of the two available genomes is quite good, but not reported in Table 1, which I would suggest. The other Oar reference genomes were published 4 and 6 years ago, with this study offering the addition of nanopore sequence.<br>Response: The scaffold L50 was added to the genome comparisons in Table 1.<br><br>Minor<br>L55 - long read vs long sequence? Do you mean contigs or scaffolds?<br>Response: The sentence refers to the increasing length of raw sequence reads that can be generated and used in genome assembly. The sentence was revised to clarify this point, and now reads, “The rapid advances in genome assembly algorithms and emergence of sequencing technologies with increasingly long reads provide the opportunity for an improved de novo assembly of the sheep reference genome.”<br><br>L233- does freebayes do polishing? This is what is suggested by the current wording

	Response: Freebayes was used in the polishing pipeline, we mention in the abstract that BCFtools is then used for consensus generation but failed to mention BCFtools in the methods section. This has been clarified "The final polishing with Illumina short read data consisted of two rounds of freebayes v1.3.1 [35] variant calling and BCFtools [36] consensus. Variants used for polishing with both Nanopolish and freebayes/BCFtools were screened with Merfin [37] which evaluates the k-mer consequences of variant calls and filters unsupported variants."<br><br>Reviewer #2: This paper is of high importance and will be well cited as people use the resource it is reporting on. It is well written and easy to read.<br>Response: Thank you to Reviewer 2 for the thorough review of this manuscript and helpful comments.<br><br>A mention of the genome length and NG50 (in addition to N50) in the abstract would be useful.<br>Response: The genome length and NG50 were added to the abstract in Line 68 and reads, "The ARS-UI_Ramb_v2.0 assembly is 2.63 Gb in length and has improved continuity (contig NG50 of 43.18 Mb) with a 19-fold and 38-fold decrease in the number of scaffolds compared with Oar_rambouillet_v1.0 and Oar_v4.0." The manuscript has also been modified to refer to NG50 rather than N50 throughout.<br><br>The authors might like to add the species name as a keyword (completely optional)<br>Response: Species name (Ovis aries) has been added as a keyword.<br><br>If not already done, the mito genome should have the start position matched to the older version. If this is already done then add it to the methods.<br>Response: Yes, the start position was matched to the Oar_rambouillet_v1.0 mitochondrial genome. This was added to the methods in Line 233. The sentence reads, "The mitochondrial genome was identified by aligning the previously annotated mitochondrial sequence from Oar_rambouillet_v1.0 (RefSeq NC_001941.1) to the assembly contigs and the start positions were matched."<br><br>I think Figure 3B should be a table not a figure part. Also Figure 5A.<br>Response: Figure 3B and Figure 5A were both separated and added as tables.
**Additional Information:**	
**Question**	**Response**
Are you submitting this manuscript to a special series or article collection?	No
**Experimental design and statistics**<br><br>Full details of the experimental design and statistical methods used should be given in the Methods section, as detailed in our Minimum Standards Reporting Checklist. Information essential to interpreting the data presented should be made available in the figure legends.<br><br>Have you included all the information	Yes

requested in your manuscript?	
**Resources**<br><br>A description of all resources used, including antibodies, cell lines, animals and software tools, with enough information to allow them to be uniquely identified, should be included in the Methods section. Authors are strongly encouraged to cite Research Resource Identifiers (RRIDs) for antibodies, model organisms and tools, where possible.<br><br>Have you included the information requested as detailed in our Minimum Standards Reporting Checklist?	Yes
**Availability of data and materials**<br><br>All datasets and code on which the conclusions of the paper rely must be either included in your submission or deposited in publicly available repositories (where available and ethically appropriate), referencing such data using a unique identifier in the references and in the “Availability of Data and Materials” section of your manuscript.<br><br>Have you have met the above requirement as detailed in our Minimum Standards Reporting Checklist?	Yes

Manuscript

Click here to access/download;Manuscript;ARS-UI_Ramb_v2.0 manuscript plus annotation final_revised.docx

An improved ovine reference genome assembly to facilitate in depth functional annotation of the sheep genome

Kimberly M. Davenport[1], Derek M. Bickhart[2], Kim C. Worley[3], Shwetha C. Murali[4], Mazdak Salavati[5], Emily L. Clark[6], Noelle E. Cockett[7], Michael P. Heaton[8], Timothy P.L. Smith[9], Brenda M. Murdoch[10]*, and Benjamin D. Rosen[11]*

[1]Department of Animal, Veterinary, and Food Sciences, University of Idaho, 875 Perimeter Dr., Moscow, ID, United States 83843. Email: kmdavenport@uidaho.edu

[2]US Dairy Forage Research Center, USDA-ARS, 1925 Linden Drive, Madison, WI, United States 53706. Email: derek.bickhart@usda.gov

[3]Baylor College of Medicine, One Baylor Plaza, Houston, TX, United States 77030. Email: kworley@bcm.edu

[4]Baylor College of Medicine, One Baylor Plaza, Houston, TX, United States 77030. Email: shwethac@gmail.com

[5]The Roslin Institute, Royal (Dick) School of Veterinary Studies, The University of Edinburgh, Easter Bush Campus, Midlothian, United Kingdom, EH25 9RG, United Kingdom. Email: mazdak.salavati@roslin.ed.ac.uk

[6]The Roslin Institute, Royal (Dick) School of Veterinary Studies, The University of Edinburgh, Easter Bush Campus, Midlothian, United Kingdom, EH25 9RG. Email: emily.clark@roslin.ed.ac.uk

[7]Utah State University, Old Main Hill, Logan, UT 84322. Email: noelle.cockett@usu.edu

[8]US Meat Animal Research Center, USDA-ARS, State Spur 18D, Clay Center, NE 68933. Email: mike.heaton@usda.gov

[9]US Meat Animal Research Center, USDA-ARS, State Spur 18D, Clay Center, NE 68933. Email: tim.smith2@usda.gov

[10]Department of Animal, Veterinary, and Food Sciences, University of Idaho, 875 Perimeter Dr., Moscow, ID 83843. Email: bmurdoch@uidaho.edu

[11]Animal Genomics and Improvement Laboratory, USDA-ARS, 10300 Baltimore Avenue, Beltsville, MD 20705. Email: ben.rosen@usda.gov

Correspondence:
Brenda M. Murdoch
bmurdoch@uidaho.edu
Benjamin D. Rosen
ben.rosen@usda.gov

Abstract

Background

The domestic sheep (*Ovis aries*) is an important agricultural species raised for meat, wool, and milk across the world. A high-quality reference genome for this species enhances the ability to discover genetic mechanisms influencing biological traits. Further, a high-quality reference genome allows for precise functional annotation of gene regulatory elements. The rapid advances in genome assembly algorithms and emergence of sequencing technologies with increasingly long reads provide the opportunity for an improved *de novo* assembly of the sheep reference genome.

Findings

Short-read Illumina (55x coverage), long-read PacBio (75x coverage), and Hi-C data from this ewe retrieved from public databases were combined with an additional 50x coverage of Oxford Nanopore data and assembled with canu v1.9. The assembled contigs were scaffolded using Hi-C data with Salsa v2.2, gaps filled with PBsuitev15.8.24, and polished with Nanopolish v0.12.5. After duplicate contig removal with PurgeDups v1.0.1, chromosomes were oriented and polished with two rounds of a pipeline which consisted of freebayes v1.3.1 to call variants, Merfin to validate them, and BCFtools to generate the consensus fasta. The ARS-UI_Ramb_v2.0 assembly is 2.63 Gb in length and has improved continuity (contig NG50 of 43.18 Mb) with a 19-fold and

38-fold decrease in the number of scaffolds compared with Oar_rambouillet_v1.0 and Oar_v4.0. ARS-UI_Ramb_v2.0 has greater per-base accuracy and fewer insertions and deletions identified from mapped RNA sequence than previous assemblies.

Conclusions

The ARS-UI_Ramb_v2.0 assembly is a substantial improvement in contiguity that will optimize the functional annotation of the sheep genome and facilitate improved mapping accuracy of genetic variant and expression data for traits in sheep.

Keywords: Rambouillet, genome assembly, reference genome, sheep, *Ovis aries*

Context

The domestic sheep (*Ovis aries*) is a globally important livestock species raised for a variety of purposes including meat, wool, and milk. Domestication likely occurred in multiple events approximately 11,000 years ago [1-4]. Selection for desirable traits including meat, wool, and milk began approximately 4,000-5,000 years ago [2,4]. Modern sheep breeds exhibit a wide variety of phenotypes and adaptations to specific environments, for example the enhanced parasite tolerance evident in hair sheep [5,6]. As many as 1,400 breeds of sheep exist today [7-9] including the Rambouillet breed developed in France from a Merino fine wool lineage that is regarded for its ability to produce high quality wool as well as meat products in production systems across the world [10,11].

Genome research in sheep holds promise to improve efficiency and sustainability of production and reduce the environmental effects of animal agriculture [12]. The first sheep reference genome assembly was based on whole genome shotgun (WGS) short-read sequencing, scaffolded by genetic linkage and radiation hybrid maps. The sequence came from two unrelated Texel breed sheep, with the first assembly draft (Oar_v3.1; International Sheep Genomics Consortium, 2010) having a contig NG50, based on a 2.6 gigabase (Gb) genome size, of 39 kilobases (kb) and the update (Oar_v4.0) [13] boosting the NG50 metric to 145 kb. More recently, the Ovine Functional Annotation of Animal Genomes (FAANG) project proposed to perform a variety of genome annotation assays for dozens of tissues from a single animal [14,15]. To maximize the success of assays that depend on mapping sequence data to a reference,

the FAANG project assembled the genome of that animal, a female of the Rambouillet breed. The assembly, released in 2017 (Oar_rambouillet_v1.0, GenBank accession GCF_002742125; Worley et al., unpublished) is based on a combination of Pacific Biosciences RSII WGS long-read and Illumina short-read sequencing. It has an improved contig NG50 of 2.9 megabases (Mb) and is generally regarded as the official reference assembly for global sheep research.

The continued maturation of long read sequencing technologies provided an opportunity to improve upon the sheep reference genome assembly. Since most of the proposed FAANG annotation assays had already been performed on the Rambouillet ewe, lung tissue from the same animal was chosen for DNA extraction. This allowed the use of existing long read data to supplement new, longer-read, Oxford Nanopore PromethION sequencing. We report a *de novo* assembly of the same Rambouillet ewe used for Oar_rambouillet_v1.0, based on approximately 50x coverage of nanopore reads (N50 47kb) and 75x coverage PacBio reads (N50 13kb). The new assembly, ARS-UI_Ramb_v2.0 offers a 15-fold improvement in contiguity and increased accuracy, providing a basis for regulatory element annotation in the FAANG project and facilitating the discovery of biological mechanisms that influence traits important in global sheep research and production.

Methods

Sampling Strategy

The fullblood Rambouillet ewe used for this genome assembly (Benz 2616, USMARC ID 200935900) (Figure 1) was selected by the Ovine Functional Annotation of Animal Genomes project and acquired from the USDA. Tissues were collected postmortem from the healthy six-year-old ewe as approved by the Utah State University Institutional Animal Care and Use Committee. A full description of the tissue collection strategy is available in the FAANG Data Coordination Center [15,16]. Details regarding the tissues collected from the animal are available under BioSample number SAMEG329607 [17].

Sequencing and Data Acquisition

DNA was extracted from approximately 50 mg of lung tissue using phenol:chloroform-based method as described (Logsdon 2019). Briefly, the frozen tissue was pulverized in a cryoPREP CP02 tissue disruption system (Covaris Inc., Woburn MA) as recommended by the manufacturer. The powdered tissue was transferred to a 50 mL conical tube and mixed in 200 µL of phosphate buffered saline (Sigma-Aldrich, St. Louis MO). The tissue was then diluted in 10 mL of buffer TLB (100mM NaCl, 10mM Tris-HCl pH 8.0, 25 mM EDTA, 0.5% SDS) and mixed by vortexing, then incubated with 20 µL 10 mg/mL RNase A at 37ºC for one hour with gentle shaking. Protein digestion was performed with 100 µL Proteinase K (20 mg/mL) at 50ºC for 2 hours, with slow rotation of the tube to mix every 30 minutes. The lysate was distributed equally into two 15 mL Phase Lock tubes (Quantabio, Beverly MA) and each tube received 5 mL of TE-saturated Phenol (Sigma-Aldrich, St. Louis MO) followed by mixing on a tube rotator at 20 RPM for 10 minutes at 22ºC. The aqueous layer was collected after separating at 2300xg

for 10 minutes and transferred to another Phase Lock tube. A second extraction performed in the same way as the first was conducted using 2.5 mL phenol and 2.5 mL chloroform:isoamyl alcohol (Sigma). The final aqueous phase was transferred to a 50 mL conical tube and the DNA precipitated with 2 mL of 5M ammonium acetate and 15 mL of ice-cold 100% ethanol. The DNA was pulled from the alcohol using a Pasteur pipet "hook" and placed in 10 mL of cold 70% ethanol to wash the pellet. The ethanol was poured off and the DNA pellet dried for 20-30 minutes, then dissolved in a dark drawer at room temperature for 48 hours in 1 mL of 10mM Tris-Cl pH 8.5. Library preparation for Oxford Nanopore long read sequencing was performed with an LSK-109 template preparation kit as recommended by the manufacturer (Oxford Nanopore, Oxford U.K.) with modifications as described by Logsdon (https://www.protocols.io/view/hmw-gdna-purification-and-ont-ultra-long-read-data-bchhit36?comment_id=88927). The ligated template was sequenced with a PromethION instrument using four R9.4 flow cells. (Oxford Nanopore Technologies, Oxford, United Kingdom). Output as fast5 files were basecalled with Guppy v3.1 [18].

Sequence data used in the previous Oar_rambouillet_v1.0 assembly was retrieved from the Sequence Read Archive listed under project number PRJNA414087 [15]. PacBio RS II sequence generated from DNA extracted from whole blood was retrieved from SRX3445660, SRX3445661, SRX3445662, and SRX3445663. The Hi-C sequence data generated from liver using HindIII enzyme and sequenced at 150 bp paired end with an Illumina HiSeq X Ten was retrieved from SRX3399085 and SRX3399086. Short read whole genome sequencing from DNA extracted from whole blood collected from the Rambouillet ewe was performed with an Illumina HiSeq X Ten sequenced at 150 bp paired end and was retrieved from SRX3405602. Further

details about these sequences can be found under the umbrella project number PRJNA414087. Short read 45 bp paired end whole genome sequence from an Illumina Genome Analyzer II generated from Texel sheep used in previous genome assemblies were retrieved from the Sequence Read Archive under accessions SRX511533-SRX511565 (BioProject PRJNA169880).

Assembly

Contigs were assembled with Oxford Nanopore and PacBio reads generated as described above using canu v1.8 through the trimmed reads stage of assembly. Parameters for contig construction were set as “batOptions=-dg 4 -db 4 -mo 1000” [19]. Canu v1.9 was used to complete the contig assembly because this update demonstrates better consensus generation of the overlapped contigs in the final step in the assembly process [20,21]. The corrected error rate option was set as “correctedErrorRate=0.105.”

Scaffolding

Two Hi-C datasets from liver tissue from two different library preparations were retrieved as described above. The Hi-C reads were first aligned to the polished contigs using the Arima Genomics mapping pipeline [22]. This pipeline first maps paired end reads individually with bwa-mem, then removes the 3’ end of reads identified as chimeric and span ligation junctions. Reads were then paired, filtered by mapping quality with samtools [23], and PCR duplicates

removed with Picard [24]. The two Hi-C libraries were merged in the final step in the Arima pipeline to generate the merged BAM file. The BAM file was converted to a BED file for input into Salsa using the bedtools command bamToBed [25]. Salsa v2.2 was used for scaffolding by implementing "python run_pipeline.py -a contigs.fasta -l contigs.fasta.fai -b alignment.bed -e HindIII -o scaffolds -m yes" [26].

The Hi-C reads were aligned to the scaffolded assembly with the Arima Genomics mapping pipeline and then processed with PretextMap to visually evaluate the scaffolds as a contact map in PretextView [27]. The scaffolded assembly was also compared to *Oar_rambouillet_v1.0* by aligning the two genomes with "minimap2 -cx asm5 Oar_rambouillet_v1.0_genomic.fasta scaffolds.fasta > alignment.paf" [28]. A dotplot of the alignment was visualized with D-Genies [29]. Scaffolds were edited based on visual inspection of the contact map and dotplot, as well as the Hi-C alignment file. Scaffold joins and rearrangements were incorporated to the assembly using the *agp2fasta* mode of CombineFasta [30].

Gap Filling and Polishing

Gap filling was completed with pbsuite v15.8.24 using both the PacBio and Oxford Nanopore reads. Nanopolish v0.12.5 [31] with the NanoGrid parallel wrapper [32] was employed with the raw fast5 files generated from the PromethION sequencing to polish the assembly. Duplicates were removed using PurgeDups v1.0.1 [33]. The chromosome orientation was confirmed in the polished assembly by identifying telomeres and centromeres using RepeatMasker v4.1.1 [34].

The mitochondrial genome was identified by aligning the previously annotated mitochondrial sequence from Oar_rambouillet_v1.0 (RefSeq NC_001941.1) to the assembly contigs and the start positions were matched. Chromosomes were oriented centromere to telomere and placed in chromosome number order. The final polishing with Illumina short read data consisted of two rounds of freebayes v1.3.1 [35] variant calling and BCFtools [36] consensus. Variants used for polishing with both Nanopolish and freebayes/BCFtools were screened with Merfin [37] which evaluates the k-mer consequences of variant calls and filters unsupported variants.

RNA Sequencing

RNA sequencing data was generated from five tissues including skin, thalamus, pituitary, lymph node (mesenteric), and abomasum pylorus collected from the animal used to assemble the reference genome. Details regarding the RNA isolation protocol, library preparation, and sequencing as well as the raw data can be found in GenBank under BioProject PRJEB35292, specifically under SRA run numbers ERR3665717 (skin), ERR3728435 (thalamus), ERR3650379 (pituitary), ERR3665711 (lymph node mesenteric), and ERR3650373 (abomasum pylorus). Reads were trimmed with Trim Galore v0.6.4 [38] and alignment to both Rambouillet genomes was performed with STAR v2.7 using default parameters [39]. Indels were identified with bcftools mpileup, filtering allele depth (AD) at > 5 [40].

Annotation

The annotation for ARS-UI_Ramb_v2.0, NCBI Ovis aries Annotation Release 104, is available in RefSeq and other NCBI genome resources (https://ftp.ncbi.nlm.nih.gov/genomes/all/annotation_releases/9940/104).

Here we also provide a liftover of the annotation for Oar_rambouillet_v1.0 onto ARS-UI_Ramb_v2.0. The annotation used for the liftover was NCBI v103 GCF_002742125.1_Oar_rambouillet_v1.0_genomic.fna.gz. The GFF3 format gene annotation file was prepared for processing using liftOff v1.5.2 [41]. A set of matching chromosome names for Oar_rambouillet_v1.0 and ARS-UI_Ramb_v2.0 were generated according to the instructions for liftOff (*paste -d "," <(cut -d' ' -f1 ramb1.chr) <(cut -d' ' -f1 ramb2.chr) > chroms.txt*). The GFF file (annotation Ramb1LO2) generated by liftOff is included in Supplementary File 1 (Ramb_v1.0_NCBI103_lifted_over_ARS-UI_Ramb_v2.0.gff.gz).

To compare the breakdown of transcripts captured by the three annotations (Oar_Rambouillet_v1.0, Ramb1LO2 (liftover) and ARS-UI_Ramb_v2.0), we generated transcript expression estimates using Kallisto v0.44.0 [42]. For the lifted over gene annotation the GFF file (Ramb_v1.0_NCBI103_lifted_over_ARS-UI_Ramb_v2.0.gff.gz) was used to generate transcriptome sequence FASTA files, as a Kallisto index, for transcript expression estimation. Briefly, exonic blocks were extracted from the GFF3 file using the awk command (*awk '($3~/exon/)' input.gff)*. The getfasta and groupby plugins from bedtools v2.30.0 [43] were used to extract the exonic sequences and group them by transcript name. Exonic sequences for each transcript were appended in the correct order, to produce the complete sequence for each transcript. The FASTA format file for the whole transcriptome was created using all of the

transcript level FASTA sequences for the liftover annotation Ramb1LO2 (Supplementary File 2; Ramb1LO2_NCBI103_geneBank_rna.fa). The set of scripts used for this step are included in Supplementary File 3. The Kallisto indices for Oar_Rambouillet_v1.0 (GCF_002742125.1_Oar_rambouillet_v1.0_rna.fna.gz), Ramb1LO2 (liftover; Ramb1LO2_NCBI103_geneBank_rna.fa) and ARS-UI_Ramb_v2.0 (GCF_016772045.1_ARS-UI_Ramb_v2.0_rna.fna.gz) were then used with the RNA-Seq data from the 61 tissues from Benz2616 (GenBank BioProject PRJNA414087 and PRJEB35292) to estimate transcript level expression for every tissue as transcript per million mapped reads (TPM) and compared across the three annotations.

Data Validation and Quality Control

Assembly Quality Statistics

The four flow cells of PromethION data produced 136 Gb of WGS sequence (approximately 51x coverage) in reads having a read N50 of 47 kb. The initial generation of contigs used this data as well as 198.1 Gb of RSII data with a read N50 of 12.9 kb. The ARS-UI_Ramb_v2.0 assembly was submitted to NCBI GenBank under accession number GCA_016772045.1, and statistics of contigs and scaffolds following initial polishing, scaffolding with Hi-C data and manual editing, gap-filling, and final polishing, are shown in Table 1. The assembly improved on the Oar_v4.0/Oar_rambouillet_v1.0 sheep reference assemblies in all continuity measures (Table 1) including a 286/17-fold increase in contig N50 (the size of the shortest contig for which all larger

contigs contain half of the total assembly), a 214/33-fold reduction in the number of contigs in the assembly and concomitant 209/13-fold reduction of contig L50 (the number of contigs making up half of the total assembly), and 38/19-fold reduction in total number of scaffolds. Manual curation of scaffolds using Hi-C data improved scaffold continuity and led to chromosome length scaffolds (Figure 2).

The Themis-ASM pipeline [44] was implemented to further assess assembly quality and compare sheep genome assemblies. Short read sequence from both the Rambouillet ewe used in this assembly and Texel sheep from previous sheep genome assemblies were used to compare ARS-UI_Ramb_v2.0 with Oar_rambouillet_v1.0 and Oar_v4.0 assemblies.

The k-mer based quality value and error rates improved with ARS-UI_Ramb_v2.0 compared with Oar_rambouillet_v1.0 and Oar_v4.0. This is also reflected in the proportion of complete assembly based on k-mers (merCompleteness), which is similar between ARS-UI_Ramb_v2.0 and Oar_rambouillet_v1.0 and both are higher than Oar_v4.0. Further, the SNP and indel quality value (baseQV) were greatest overall in ARS-UI_Ramb_v2.0 (41.84), followed by Oar_rambouillet_v1.0 (40.69) and Oar_v4.0 (32.40). The percentage of short reads not mapped to the genome was ≤1% in all three assemblies.

The completeness of ARS-UI_Ramb_v2.0 was evaluated by examining the presence or absence of evolutionarily conserved genes in each assembly using Benchmarking Universal Single-Copy Ortholog (BUSCO) scores generated as an output of the Themis-ASM pipeline. The percent of single copy complete BUSCOs were higher (90.7%) in ARS-UI_Ramb_v2.0 when compared

with Oar_rambouillet_v1.0 (90.1%) and Oar_v4.0 (86.1%). Complete duplicated BUSCO percentage was highest in Oar_rambouillet_v1.0 (1.6%) compared with ARS-UI_Ramb_v2.0 (1.4%), and lowest in Oar_v4.0 (1.0%). Further, ARS-UI_Ramb_v2.0 had the lowest percent of fragmented and missing BUSCOs (2.0% and 5.9%, respectively) compared with Oar_rambouillet_v1.0 (2.1% and 6.2%, respectively) and Oar_v4.0 (3.7% and 9.2%, respectively).

The three sheep genome assemblies were also compared with a feature response curve in which the quality of the assembly is analyzed as a function of the features, or maximum number of possible errors, allowed in the contigs (Figure 3) [45]. Both the ARS-UI_Ramb_v2.0 and Oar_v4.0 feature response curves peak higher and to the left of Oar_rambouillet_v1.0, which indicate fewer errors in these assemblies (Figure 3A). The ARS-UI_Ramb_v2.0 genome also has fewer regions with either low or high coverage overall and for paired reads, suggesting fewer coverage issues, as well as fewer improperly paired or unmapped single reads when compared with other assemblies (Table 2). The number of high Comp/Expansion (CE) statistics in ARS-UI_Ramb_v2.0 was intermediate between Oar_rambouillet_v1.0 (higher) and Oar_v4.0 (lower), however this latest assembly had the lowest number of regions with low CE statistics.

Comparative alignment of ARS-UI_Ramb_v2.0 with previous assemblies Oar_rambouillet_v1.0 and Oar_v4.0 and visualization with a dotplot revealed a high amount of agreement between assemblies (Figure 4). Interestingly, chromosome 11 was improperly oriented in Oar_rambouillet_v1.0, and after confirming centromere and telomere locations on this chromosome, this was resolved in the ARS-UI_Ramb_v2.0 assembly. The percent identity

between ARS-UI_Ramb_v2.0 is very high when compared with Oar_rambouillet_v1.0 which was expected considering the same animal was used in both assemblies. However, Oar_v4.0 was assembled from Texel sheep, which is apparent in the percent identity in the dotplot.

In summary, ARS-UI_Ramb_v2.0 offers greater contiguity, improved quality, more complete BUSCOs, and fewer assembly errors when compared with previous assemblies.

RNA sequencing alignment

Insertions and deletions (indels) in the ARS-UI_Ramb_v2.0 assembly were characterized and compared with Oar_rambouillet_v1.0 by mapping 150 bp paired-end RNA-seq data from skin, thalamus, pituitary, lymph node (mesenteric), and abomasum pylorus generated from the same animal used to assemble the reference genome (Table 3). In all five tissues, ARS-UI_Ramb_v2.0 had nearly half of the number of indels compared with Oar_rambouillet_v1.0. Most indels identified in both assemblies were 1bp in length. The ARS-UI_Ramb_v2.0 had a greater number of uniquely mapped reads in each tissue when compared with Oar_rambouillet_v1.0, leading to an approximate 2% increase in the percent of uniquely mapped reads in most tissues except pituitary, which saw an almost 13% improvement. The number of reads that mapped to multiple loci decreased in the new assembly by 12.59% in pituitary, and 1-2% in other tissues. Further, ARS-UI_Ramb_v2.0 had fewer unmapped reads than Oar_rambouillet_v1.0 across all five tissues by an average of 0.15%.

Annotation

The ARS-UI_Ramb_v2.0 annotation represents a substantial improvement over the annotation on Oar_rambouillet_v1.0. For example, for ARS-UI_Ramb_v2.0 16,500 coding genes have an ortholog to human (compared to 16,319 for Oar_rambouillet_v1.0), and the BUSCO scores demonstrate that 99.1% of the gene models (cetartiodactyla_odb10) are complete in the new annotation versus 98.8% in the previous one. The annotation for ARS-UI_Ramb_v2.0 includes Iso-Sequencing for 8 tissues to improve contiguity of gene models, and CAGE sequencing for 56 tissues to define TSS, that were not used to annotate Oar_rambouillet_v1.0. The full report for the annotation release is available at: (https://www.ncbi.nlm.nih.gov/genome/annotation_euk/Ovis_aries/104).

Using Kallisto we compared the number of expressed transcripts, for the RNA-Seq dataset of 61 tissue samples from Benz2616, across the three annotations (Oar_Rambouillet_v1.0, Ramb1LO2 (liftover) and ARS-UI_Ramb_v2.0). There was a considerable increase in the number of transcripts captured by the annotation for ARS-UI_Ramb_v2.0 (60,064) relative to Oar_Rambouillet_v1.0 (42,058) and the liftover annotation (Ramb1LO2) (40,910) (Table 4 and Figure 5). This equates to approximately 20,000 new annotated gene models for ARS-UI_Ramb_v2.0 and further reflects the substantial improvement over the annotation for Oar_Rambouillet_v1.0.

The lifted over annotation we have generated will provide a resource for those who wish to compare their results for ARS-UI_Ramb_v2.0 to previous work using Oar_Rambouillet_v1.0. Only 2.7% of protein coding transcripts were lost (1148) lifting over the

annotation for Oar_Rambouillet_v1.0 onto ARS-UI_Ramb_v2.0. According to the annotation report provided by NCBI (https://www.ncbi.nlm.nih.gov/genome/annotation_euk/Ovis_aries/104/), 70% of the annotations were identical or had only minor changes between and Oar_Rambouillet_v1.0 and ARS-UI_Ramb_v2.0.

Re-use potential

The ARS-UI_Ramb_v2.0 genome assembly serves as a reference for genetic investigation of traits important in sheep research and production across the world. This genome is assembled from the same animal used in the Ovine FAANG Project, which provides a high-quality basis for epigenetic annotation to serve the international sheep genomics community and scientific community at large.

Availability of supporting data

The data sets supporting the results of this article are available in the GenBank repository, GCA_016772045.1.

Additional files

Supplementary File 1 – Ramb_v1.0_NCBI103_lifted_over_ARS-UI_Ramb_v2.0.gff.gz

Supplementary File 2 – Ramb1LO2_NCBI103_geneBank_rna.fa

Supplementary File 3 – Supplementary_File_3_scripts.txt

Author contributions

BMM, TPLS, DMB, and BDR conceptualized the study. BMM, NEC, MPH, and TPLS selected the animal and collected samples. KW and SCM facilitated the generation of RSII, short read, and Hi-C data. TPLS facilitated the nanopore long read data generation. KMD, DMB, TPLS, BMM, and BDR performed the genome assembly, scaffolding, RNA-sequencing alignment, polishing, and quality control. MS and ELC contributed the section describing the LiftOff annotation and comparative analysis of transcript expression estimates for the three annotations. KMD, DMB, TPLS, BMM, and BDR generated tables and figures and drafted the manuscript. KMD, DMB, KW, SCM, NEC, TPLS, BMM, and BDR edited the manuscript. All authors contributed to the article and approved the final version.

Acknowledgements

The authors thank Dr. Kristen Kuhn for technical support and Dr. Kreg Leymaster for overseeing the acquisition, animal care and housing, and interstate transportation of the Rambouillet ewe.

Funding

Funding was provided by Agriculture and Food Research Initiative Competitive grants from the USDA National Institute of Food and Agriculture supporting improvements of the sheep genomes (2013-67015-21228) and FAANG activities (2013-67015-21372, 2017-67016-26301). Additional funding was received from the International Sheep Genome Consortium (217201191442) and infrastructure support from a grant to R. Gibbs from the NIH NHGRI Large-Scale Sequencing Program (U54 HG003273).

DMB was supported by appropriated USDA CRIS project 5090-31000-026-00-D. TPLS was supported by appropriated USDA CRIS Project 3040-31000-100-00D. BDR was supported by appropriated USDA CRIS Project 8042-31000-001-00-D. The USDA does not endorse any products or services. Mentioning of trade names is for information purposes only. The USDA is an equal opportunity employer.

References

1. Pedrosa S, Uzun M, Arranz JJ, Gutiérrez-Gil B, San Primitivo F, Bayón Y. Evidence of three maternal lineages in Near Eastern sheep supporting multiple domestication events. Proc Biol Sci. 2005;272:2211-7.

2. Zeder MA. Domestication and early agriculture in the Mediterranean Basin: origins, diffusion, and impact. Proc Natl Acad Sci USA. 2008;105:11597-604.

3. Chessa B, Pereira F, Arnaud F, Amorim A, Goyache F, Mainland I, Kao RR, Pemberton JM, Beraldi D, Stear MJ, Alberti A, Pittau M, Iannuzzi L, Banabazi MH, Kazwala RR, Zhang YP, Arranz JJ, Ali BA, Wang Z, Uzun M, Dione MM, Olsaker I, Holm LE, Saarma U, Ahmad S, Marzanov N, Eythorsdottir E, Holland MJ, Ajmone-Marsan P, Bruford MW, Kantanen J, Spencer TE, Palmarini M. Revealing the history of sheep domestication using retrovirus integrations. Science. 2009;324:532-6.

4. Kijas JW, Lenstra JA, Hayes B, Boitard S, Porto Neto LR, San Cristobal M, Servin B, McCulloch R, Whan V, Gietzen K, Paiva S, Barendse W, Ciani E, Raadsma H, McEwan J, Dalrymple B, International Sheep Genomics Consortium Members. Genome-wide analysis of the world's sheep breeds reveals high levels of historic mixture and strong recent selection. PLoS Biol. 2012;doi:10.1371/journal.pbio.1001258.

5. Burke JM, Miller JE. Relative resistance of Dorper crossbred ewes to gastrointestinal nematode infection compared with St. Croix and Katahdin ewes in the southeastern United States. Vet Parasitol. 2002;109:265-75.

6. Bowdridge SA, Zajac AM, Notter DR. St. Croix sheep produce a rapid and greater cellular immune response contributing to reduced establishment of *Haemonchus contortus*. Vet Parasitol. 2015;208:204-10.

7. Scherf BD. World watch list for domestic animal diversity. 3rd ed. Rome: Food and Agriculture Organization of the United Nations; 2000.

8. Lv FH, Agha S, Kantanen J, Colli L, Stucki S, Kijas JW, Joost S, Li MH, Ajmone Marsan P. Adaptations to climate-mediated selective pressures in sheep. Mol Biol Evol. 2014;31:3324-43.

9. Cao YH, Xu SS, Shen M, Chen ZH, Gao L, Lv FH, Xie XL, Wang XH, Yang H, Liu CB, Zhou P, Wan PC, Zhang YS, Yang JQ, Pi WH, Hehua E, Berry DP, Barbato M, Esmailizadeh A, Nosrati M, Salehian-Dehkordi H, Dehghani-Qanatqestani M, Dotsev AV, Deniskova TE, Zinovieva NA, Brem G, Štěpánek O, Ciani E, Weimann C, Erhardt G, Mwacharo JM, Ahbara A, Han JL, Hanotte O, Miller JM, Sim Z, Coltman D, Kantanen J, Bruford MW, Lenstra JA, Kijas J, Li MH. Historical Introgression from Wild Relatives Enhanced Climatic Adaptation and Resistance to Pneumonia in Sheep. Mol Biol Evol. 2021;38:838-55.

10. Dickinson WF, Lush JL. Inbreeding and the genetic history of the Rambouillet sheep in America. J Hered. 1933;24:19-33.

11. Zhang L, Mousel MR, Wu X, Michal JJ, Zhou X, Ding B, Dodson MV, El-Halawany NK, Lewis GS, Jiang Z. Genome-wide genetic diversity and differentially selected regions among

Suffolk, Rambouillet, Columbia, Polypay, and Targhee sheep. PLoS One. 2013;doi: 10.1371/journal.pone.0065942.

12. Rexroad C, Vallet J, Matukumalli LK, Reecy J, Bickhart D, Blackburn H, Boggess M, Cheng H, Clutter A, Cockett N, Ernst C, Fulton JE, Liu J, Lunney J, Neibergs H, Purcell C, Smith TPL, Sonstegard T, Taylor J, Telugu B, Eenennaam AV, Tassell CPV, Wells K. Genome to Phenome: Improving Animal Health, Production, and Well-Being - A New USDA Blueprint for Animal Genome Research 2018-2027. Front Genet. 2019;10:327.

13. Jiang Y, Xie M, Chen W, Talbot R, Maddox JF, Faraut T, Wu C, Muzny DM, Li Y, Zhang W, Stanton JA, Brauning R, Barris WC, Hourlier T, Aken BL, Searle SMJ, Adelson DL, Bian C, Cam GR, Chen Y, Cheng S, DeSilva U, Dixen K, Dong Y, Fan G, Franklin IR, Fu S, Guan R, Highland MA, Holder ME, Huang G, Ingham AB, Jhangiani SN, Kalra D, Kovar CL, Lee SL, Liu W, Liu X, Lu C, Lv T, Mathew T, McWilliam S, Menzies M, Pan S, Robelin D, Servin B, Townley D, Wang W, Wei B, White SN, Yang X, Ye C, Yue Y, Zeng P, Zhou Q, Hansen JB, Kristensen K, Gibbs RA, Flicek P, Warkup CC, Jones HE, Oddy VH, Nicholas FW, McEwan JC, Kijas J, Wang J, Worley KC, Archibald AL, Cockett N, Xu X, Wang W, Dalrymple BP. The sheep genome illuminates biology of the rumen and lipid metabolism. Science. 2014;344:1168-1173.

14. Murdoch BM. The functional annotation of the sheep genome project. J Anim Sci. 2019;97:16.

15. Salavati M, Caulton A, Clark R, Gazova I, Smith TPL, Worley KC, Cockett NE, Archibald AL, Clarke SM, Murdoch BM, Clark EL. Global Analysis of Transcription Start Sites in the New Ovine Reference Genome (*Oar rambouillet v1.0*). Front Genet. 2020;11:580580.

16. FAANG Data Coordination Center. 2016. https://data.faang.org/api/fire_api/samples/USU_SOP_Ovine_Benz2616_Tissue_Collection_20160426.pdf.

17. European Bioinformatics Institute, BioSample SAMEG329607. 2016. https://www.ebi.ac.uk/biosamples/samples/SAMEG329607.

18. Guppy (2021). Guppy basecaller (Version 3.1) www.nanoporetech.com.

19. Koren S, Walenz BP, Berlin K, Miller JR, Bergman NH, Phillippy AM. Canu: scalable and accurate long-read assembly via adaptive *k*-mer weighting and repeat separation. Genome Res. 2017;27:722-36.

20. Heaton MP, Smith TPL, Bickhart DM, Vander Ley BL, Kuehn LA, Oppenheimer J, Shafer WR, Schuetze FT, Stroud B, McClure JC, Barfield JP, Blackburn HD, Kalbfleisch TS, Davenport KM, Kuhn KL, Green RE, Shapiro B, Rosen BD. A Reference Genome Assembly of Simmental Cattle, Bos taurus taurus. J Hered. 2021;112:184-91.

21. Oppenheimer J, Rosen BD, Heaton MP, Vander Ley BL, Shafer WR, Schuetze FT, Stroud B, Kuehn LA, McClure JC, Barfield JP, Blackburn HD, Kalbfleisch TS, Bickhart DM, Davenport KM, Kuhn KL, Green RE, Shapiro B, Smith TPL. A Reference Genome Assembly of American Bison, Bison bison bison. J Hered. 2021;112:174-183.

22. Arima Genomics Mapping Pipeline (2019). ArimaGenomics https://github.com/ArimaGenomics/mapping_pipeline.

23. Li H, Handsaker B, Wysoker A, Fennell T, Ruan J, Homer N, Marth G, Abecasis G, Durbin R; 1000 Genome Project Data Processing Subgroup. The Sequence Alignment/Map format and SAMtools. Bioinformatics. 2009;25:2078-9.

24. PicardTools (2019). Picard Toolkit, Broad Institute (Version 2.9.2) http://broadinstitute.github.io/picard.

25. Quinlan AR. BEDTools: The Swiss-Army Tool for Genome Feature Analysis. Bioinformatics. 2014;47:1-34.

26. Ghurye J, Pop M, Koren S, Bickhart D, Chin CS. Scaffolding of long read assemblies using long range contact information. BMC Genomics. 2017;18:527.

27. Yardımcı GG, Noble W. Software tools for visualizing Hi-C data. Genome Biol. 2017;18:26.

28. Heng L. Minimap2: pairwise alignment for nucleotide sequences. Bioinformatics. 2018;34:3094-100.

29. D-Genies (2018). D-Genies (Version 1.2.0) https://github.com/genotoul-bioinfo/dgenies/releases/tag/v1.2.0.

30. CombineFasta agp2fasta (2020). CombineFasta (Version 0.0.17) https://github.com/njdbickhart/CombineFasta.

31. Loman N, Quick J Simpson J. A complete bacterial genome assembled de novo using only nanopore sequencing data. Nat Methods. 2015;12:733-735.

32. NanoGrid (2018). NanoGrid https://github.com/skoren/NanoGrid.

33. Guan D, McCarthy SA, Wood J, Howe K, Wang Y, Durbin R. Identifying and removing haplotypic duplication in primary genome assemblies. Bioinformatics. 2020;36:2896-8.

34. Smit AFA, Hubley R, Green P. RepeatMasker Open-4.0. 2013-2015. https://www.repeatmasker.org.

35. Garrison E, Marth G. Haplotype-based variant detection from short-read sequencing. arXiv preprint. 2012:1207.3907.

36. Danecek, P., Bonfield, J.K., Liddle, J., Marshall, J., Ohan, V., Pollard, M.O., Whitwham, A., Keane, T., McCarthy, S.A., Davies, R.M., Li, H.. Twelve years of SAMtools and BCFtools. Gigascience. 2021;10(2):giab008. doi:10.1093/gigascience/giab008

37. Merfin (2021). Merfin https://github.com/arangrhie/merfin.

38. Trim Galore (2020). TrimGalore (Version 0.6.6) https://www.bioinformatics.babraham.ac.uk/projects/trim_galore/.

39. Dobin A, Davis CA, Schlesinger F, Drenkow J, Zaleski C, Jha S, Batut P, Chaisson M, & Gingeras TR. STAR: ultrafast universal RNA-seq aligner. Bioinformatics. 2013;29:15-21.

40. Li H. A statistical framework for SNP calling, mutation discovery, association mapping and population genetical parameter estimation from sequencing data. Bioinformatics. 2011;27:2987-93.

41. Shumate, A., and Salzberg, S.L. (2020). Liftoff: accurate mapping of gene annotations. *Bioinformatics*. Doi:10.1093/bioinformatics/btaa1016.

42. Bray, N. L., Pimentel, H., Melsted, P., and Pachter, L. (2016). Near-optimal probabilistic RNA-seq quantification. Nat. Biotechnol. 34, 525–527. doi:10.1038/nbt.3519.

43. Quinlan, A. R., and Hall, I. M. (2010). BEDTools: A flexible suite of utilities for comparing genomic features. Bioinformatics 26, 841–842. doi:10.1093/bioinformatics/btq033.

44. Themis-ASM (2020). Themis-ASM pipeline https://github.com/njdbickhart/Themis-ASM.

45. Vezzi F, Narzisi G, Mishra B. Reevaluating Assembly Evaluations with Feature Response Curves: GAGE and Assemblathons. PLoS ONE. 2012;7:e52210.

46. Rhie A, Walenz BP, Koren S, Phillippy AM. Merqury: reference-free quality, completeness, and phasing assessment for genome assemblies. Genome Biol. 2020;21:245.

47. Koren S, Rhie A, Walenz BP, Dilthey AT, Bickhart DM, Kingan SB, Hiendleder S, Williams JL, Smith TPL, Phillippy AM. De novo assembly of haplotype-resolved genomes with trio binning. Nat Biotechnol. 2018;10.1038/nbt.4277.

Tables

Table 1: Assembly quality statistics comparison

Assembly Statistic	ARS-UI_Ramb_v2.0	Oar_rambouillet_v1.0	Oar_v4.0	Description
**Total Length (Mb)**	2628.15	2869.91	2615.52	Assembly length in Mbp
**Contig Number**	226	7,486	48,482	Total number of contigs
**Contig NG50 (bp)**	43,178,051	2,850,956	145,655	Half the length of the genome is in contigs of this size or greater, based on a 2600 Mb genome
**Contig LG50 (number of contigs)**	24	263	5,206	The smallest number of contigs whose length sum make up half of the assembly size
**Scaffold Number**	142	2,641	5,466	Total number of scaffolds and unplaced contigs in the assembly
**Scaffold L50 (number of scaffolds)**	8	8	8	The smallest number of scaffolds whose length sum make up half of the assembly size.
**merQV**	44.7721*	32.1705*	31.9131**	Kmer based quality from Merqury, which estimates the frequency of consensus errors in the assembly [46]
**merErrorRate**	0.000033327*	0.00060662*	0.000643714**	Kmer based error rate from Merqury, which estimates error rate of the assembly based on errors in kmers [46]
**merCompleteness**	93.0479*	93.4711*	92.2182**	Proportion of complete assembly estimated by Merqury based on "reliable" kmers, or kmers unlikely to be caused by sequencing error [46]
**baseQV**	41.84*	40.69*	32.40**	SNP and INDEL quality value estimated from short read data mapped to the assembly [47]
**Unmap%**	0.96*	1.00*	0.73**	Percentage of short reads that are unmapped to each assembly [47]
**COMPLETESC**	90.7	90.1	86.1	Percent of complete, single copy BUSCOs
**COMPLETEDUP**	1.4	1.6	1.0	Percent of complete, duplicated BUSCOs
**FRAGMENT**	2.0	2.1	3.7	Percent of fragmented BUSCOs
**MISSING**	5.9	6.2	9.2	Percent of missing BUSCOs

*Short read sequencing from the Rambouillet ewe used to assemble both ARS-UI_Ramb_v2.0 and Oar_rambouillet_v1.0 was used in these quality values.

**Short read sequencing from the Texel animal used to assemble Oar_v4.0 was used in these quality values.

Table 2: Specific feature counts for each genome and descriptions.

Features	ARS-UI_Ramb_v2.0	Oar_rambouillet_v1.0	Oar_v4.0	Description
**LOW_COV_PE**	7212	95166	89103	Low read coverage areas
**LOW_NORM_COV_PE**	2990	24381	26860	Low coverage of normal paired end reads
**HIGH_SPAN_PE**	6522	22628	33232	Regions with high numbers of inter-contig paired end reads
**HIGH_COV_PE**	2051	3630	26276	Regions with high read coverage
**HIGH_NORM_COV_PE**	2366	2633	1875	Regions with high coverage of normal paired end reads
**HIGH_OUTIE_PE**	2514	28766	37495	Regions with high counts of improperly paired reads
**HIGH_SINGLE_PE**	0	0	0	Regions with high counts of single unmapped reads
**STRECH_PE**	74	84	67	Regions with high Comp/Expansion (CE) statistics
**COMPR_PE**	87	92	44	Regions with low Comp/Expansion (CE) statistics

Tissue	Genome*	# input reads	# reads uniquely mapped	% of reads uniquely mapped	# reads multi-mapped	% reads multi-mapped	# reads unmapped	% reads unmapped	# indels
Skin	v2.0	62,630,134	53,990,480	86.20%	6,684,213	10.67%	1,955,441	3.12%	962
	v1.0		52,523,732	83.86%	8,114,599	12.96%	1,991,803	3.18%	2,512
	Δ	N/A	1,466,748	2.34%	-1,430,386	-2.29%	-36,362	-0.06%	-1,550
Thalamus	v2.0	54,655,873	45,721,452	83.65%	5,414,620	9.91%	3,519,801	6.44%	649
	v1.0		44,904,096	82.16%	6,126,363	11.21%	3,625,414	6.63%	1,054
	Δ	N/A	817,356	1.49%	-711,743	-1.30%	-105,613	-0.19%	-405
Pituitary	v2.0	43,368,663	39,710,031	91.56%	2,405,103	5.55%	1,253,529	2.89%	604
	v1.0		34,115,417	78.66%	7,866,251	18.14%	1,386,995	3.20%	960
	Δ	N/A	5,594,614	12.90%	-5,461,148	-12.59%	-133,466	-0.31%	-356
Lymph node – mesenteric	v2.0	43,673,576	38,819,419	88.88%	3,562,121	8.16%	1,292,036	2.96%	684
	v1.0		38,296,065	87.69%	4,057,915	9.29%	1,319,596	3.02%	999
	Δ	N/A	523,354	1.19%	-495,794	-1.13%	-27,560	-0.06%	-315
Abomasum pylorus	v2.0	45,977,534	41,018,529	89.21%	2,978,042	6.48%	1,980,963	4.31%	512
	v1.0		40,403,981	87.88%	3,533,015	7.68%	2,040,538	4.44%	846
	Δ	N/A	614,548	1.33%	-554,973	-1.20%	-59,575	-0.13%	-334

Table 3: RNA-seq alignment statistics to ARS-UI_Ramb_v2.0 and Oar_rambouillet_v1.0 from five different tissues.

* Genomes include v2.0 (ARS-UI_Ramb_v2.0) and v1.0 (Oar_rambouillet_v1.0) and the difference (Δ).

Table 4: Expressed transcripts (TPM>0) in Benz2616 tissues (n=61) based on Oar_rambouillet_v1.0 and ARS-UI_Ramb_v2.0 and lift over (LO) (RefSeq v103 & 104, respectively).

**Gene Biotype**	**Oar_rambouillet_v1.0**	**Oar_rambouillet_v1.0 LO**	**ARS-UI_Ramb_v2.0**	**LO vs. Oar_rambouillet_v1.0**	**LO vs. ARS-UI_Ramb_v2.0**	**Oar_rambouillet_v1.0 vs. ARS-UI_Ramb_v2.0**
Guide RNA	30	29	30	-1	-1	0
lncRNA	3929	3752	6018	-177	-2266	-2089
Protein coding	42058	40910	60064	-1148	-19154	-18006
rRNA	272	17	22	-255	-5	250
snoRNA	644	590	593	-54	-3	51
snRNA	997	907	879	-90	28	118

Figure Legends

Figure 1: Image of Benz 2616 Rambouillet ewe selected for the ovine reference genome assembly.

Figure 2: Hi-C contact map comparison of ARS-UI_Ramb_v2.0 A) directly after scaffolding and before manual curation and B) after manual curation with scaffold rearrangements and joins.

Figure 3: Assembly error comparison between ARS-UI_Ramb_v2.0, Oar_rambouillet_v1.0, and Oar_v4.0 in a feature response curve displaying sorted lengths of the assemblies with the fewest errors.

Figure 4: Dotplot comparison of genome assemblies between A) ARS-UI_Ramb_v2.0 and Oar_rambouillet_v1.0, and B) ARS-UI_Ramb_v2.0 and Oar_v4.0.

Figure 5: Kallisto comparison of the number of expressed transcripts for the RNA-Seq dataset of 61 tissue samples from Benz2616, across the three annotations (Oar_rambouillet_v1.0, Ramb1LO2 (liftover) and ARS-UI_Ramb_v2.0).

Click here to access/download;Figure;Figure1.jpg

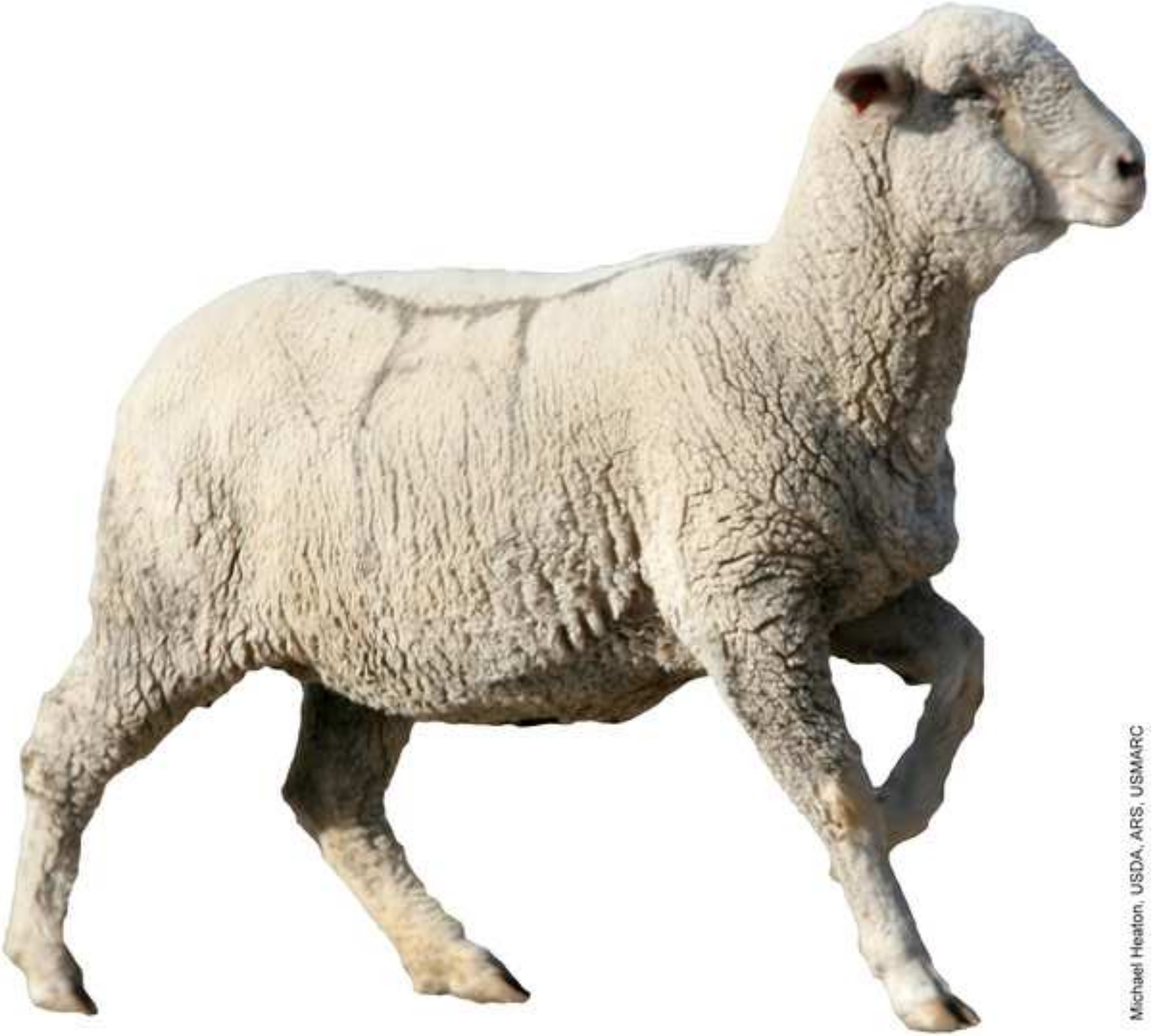

Figure2 Click here to access/download;Figure;Figure2.jpg

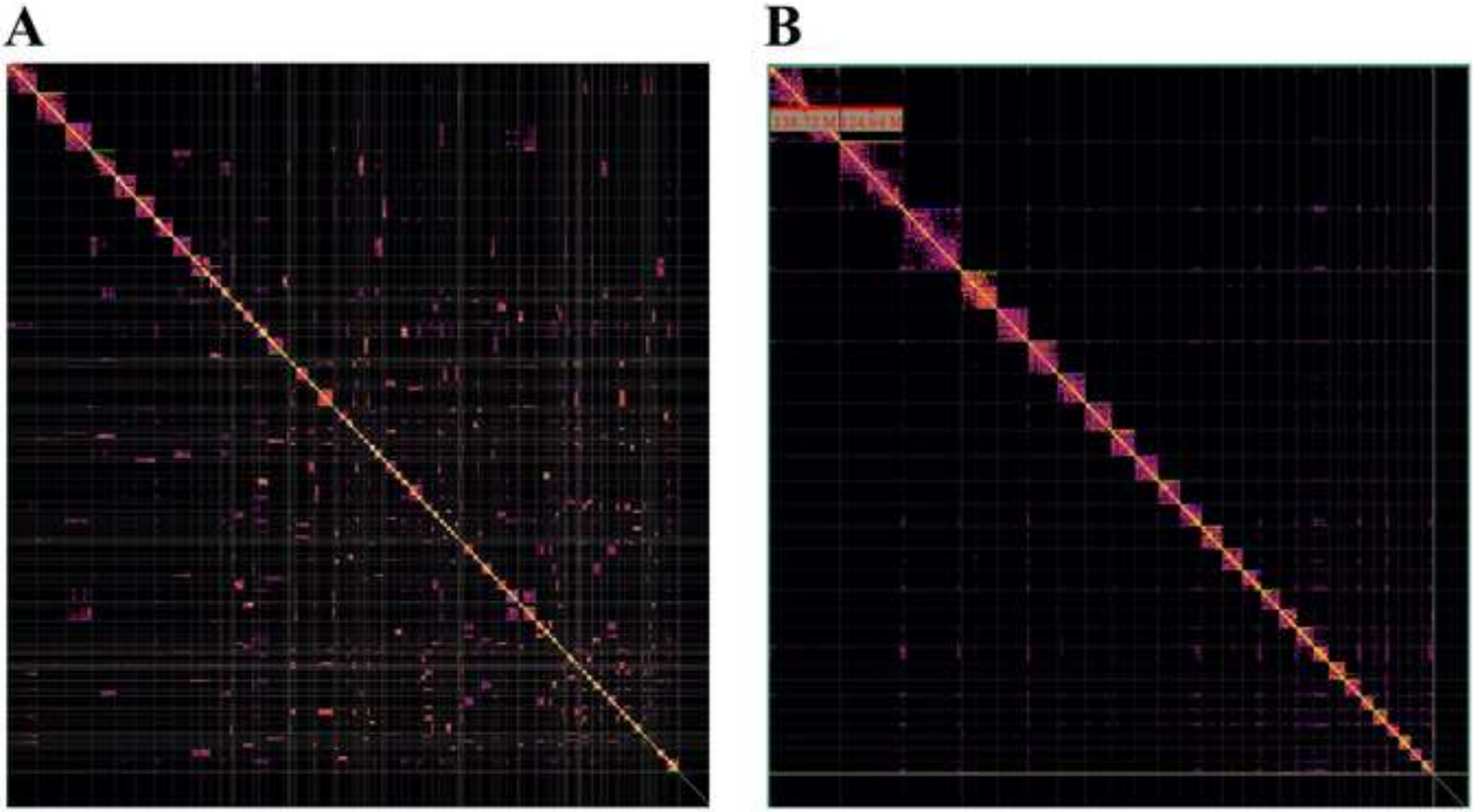

Figure3

Click here to access/download;Figure;Figure3.jpg

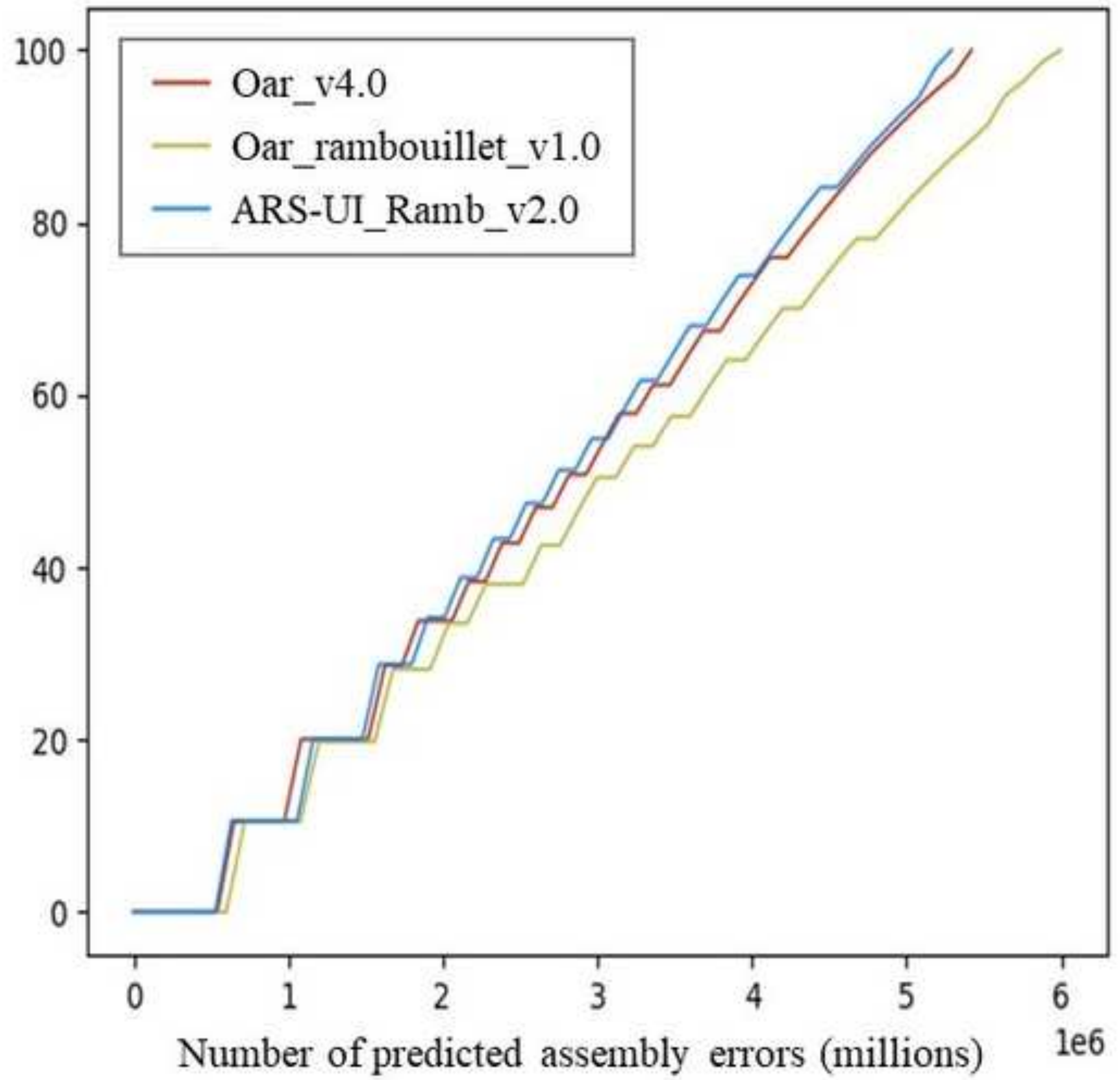

A

B

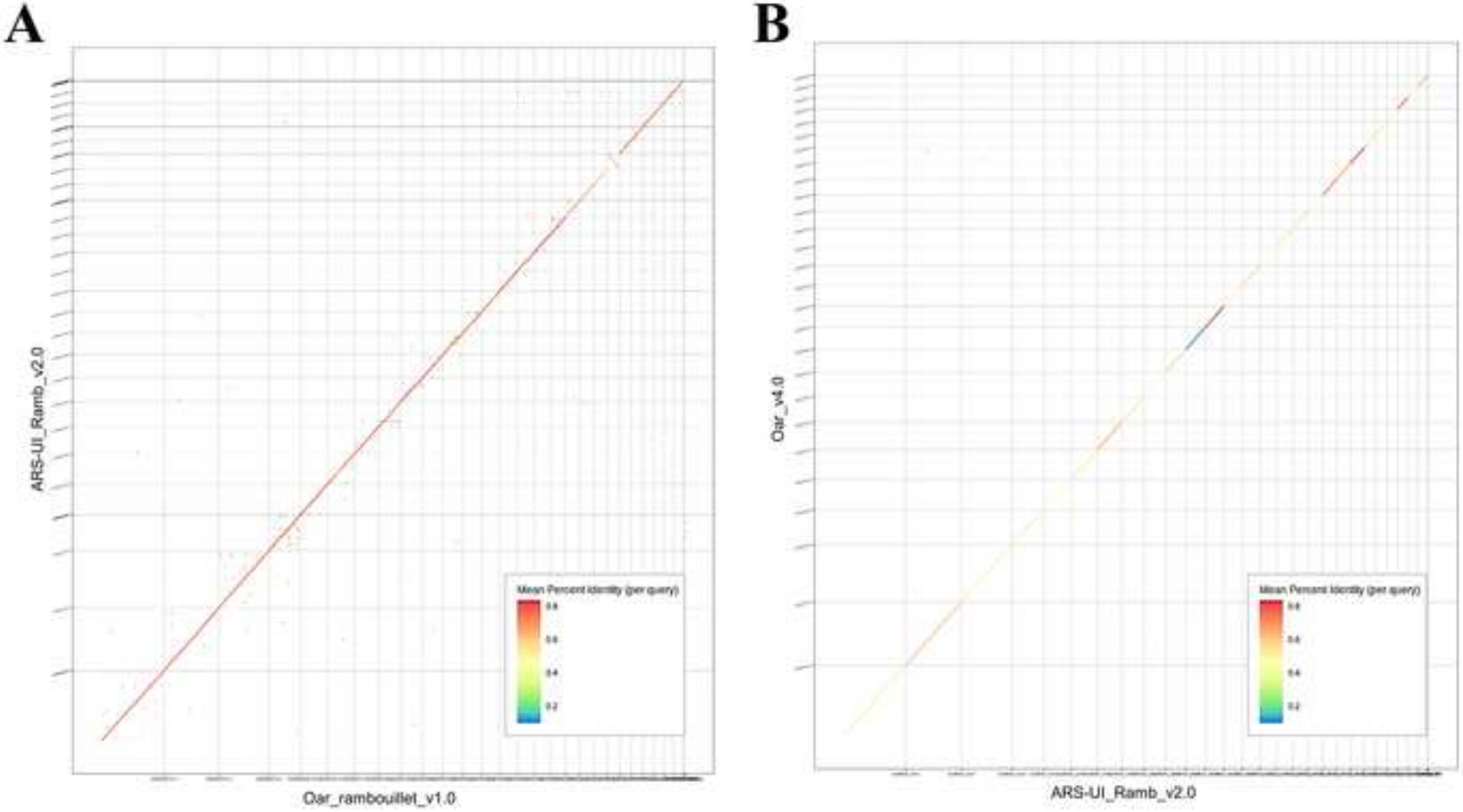
ARS-UI_Ramb_v2.0
Oar_rambouillet_v1.0
Mean Percent Identity (per query)
Oar_v4.0
ARS-UI_Ramb_v2.0
Mean Percent Identity (per query)

Figure5

Click here to access/download;Figure;Figure5.png

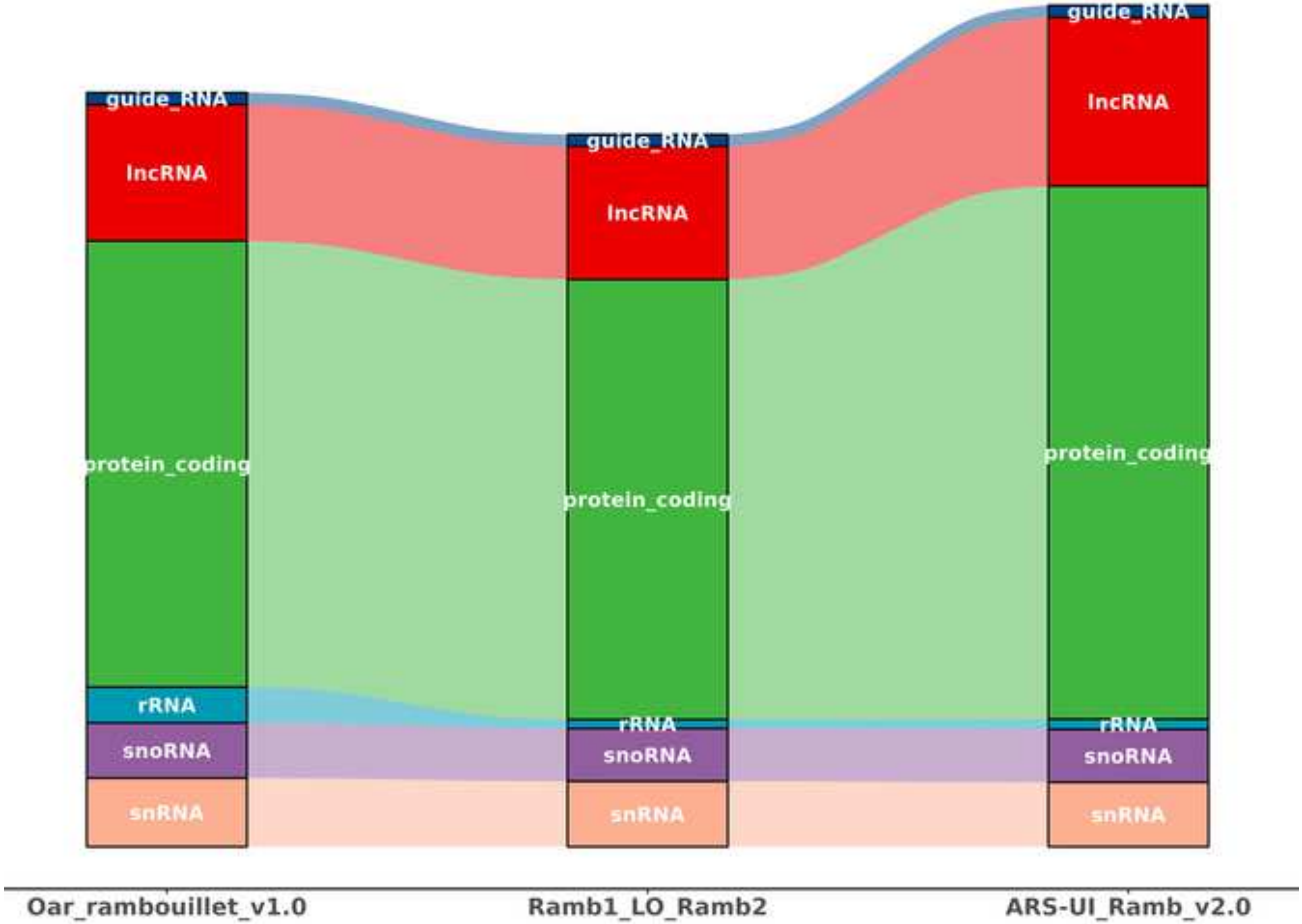

Supplement: giab096_GIGA-D-21-00165_Revision_1 [file giab096_giga-d-21-00165_revision_1.pdf]
