# Supplementary material for: An improved ovine reference genome assembly to facilitate in-depth functional annotation of the sheep genome
Source: Gigascience. 2022 Feb 4;11:giab096. doi: 10.1093/gigascience/giab096 (PMC8848310; doi:10.1093/gigascience/giab096)
Supplement: giab096_Supplemental_Files [file giab096_supplemental_files.zip › Supplementary File 3.docx]

#Reconstructing the transcriptome FASTA sequence for the lifted over gene annotation

#Tools used

module load pigz/2.3.3

module load BEDTools/2.30.0

module load samtools/1.9

module load kallisto/0.44.0

#Extracting the exonic block from the GFF file

zcat Ramb_v1.0_NCBI103_lifted_over_ARS-UI_Ramb_v2.0.gff.gz | \

awk '($3~/exon/)' | \

pigz > Ramb1LO2_NCBI103_exons.gff.gz

#Creating a modified GFF3 file format

paste <(zcat Ramb1LO2_NCBI103_exons.gff.gz | cut -f1-2) \

<(zcat Ramb1LO2_NCBI103_exons.gff.gz | cut -f9 | cut -d";" -f2| sed 's/Parent\=rna-//g') \

<(zcat Ramb1LO2_NCBI103_exons.gff.gz | cut -f4- ) > Ramb1LO2.gff

#Coversion to BED6 format

awk '{OFS="\t"; print $1,$4,$5,$3,0,$7}' Ramb1LO2.gff > Ramb1LO2.bed

#Example output

CM028704.1 42238 42395 XM_027962292.1 0 -

CM028704.1 42690 43127 XM_027962292.1 0 -

CM028704.1 43130 43378 XM_027962292.1 0 -

CM028704.1 43381 43588 XM_027962292.1 0 -

CM028704.1 43591 43756 XM_027962292.1 0 -

CM028704.1 43853 44085 XM_027962292.1 0 -

CM028704.1 45265 45335 XM_027962292.1 0 -

CM028704.1 46081 46232 XM_027962292.1 0 -

CM028704.1 46503 46709 XM_027962292.1 0 -

CM028704.1 74992 75652 XR_003588699.1 0 -

CM028704.1 76859 78063 XR_003588699.1 0 -

CM028704.1 78522 79261 XR_003588700.1 0 +

CM028704.1 79410 79494 XR_003588700.1 0 +

CM028704.1 147143 147427 XM_027962305.1 0 -

CM028704.1 147429 148122 XM_027962305.1 0 -

CM028704.1 148124 148170 XM_027962305.1 0 -

CM028704.1 148172 148597 XM_027962305.1 0 -

CM028704.1 150156 150304 XM_027962305.1 0 -

CM028704.1 158201 158296 XM_027964169.1 0 +

CM028704.1 164690 165052 XM_027964169.1 0 +

CM028704.1 165371 165532 XM_027964169.1 0 +

CM028704.1 166287 166321 XM_027964169.1 0 +

CM028704.1 166520 167538 XM_027964169.1 0 +

#Verifying the collapse of exon to transcript models and uniqueness

#Exons were grouped by transcript and counted per group (should be 1 == score column in the output bed)

#After sorting by the transcript id (-k 4,4) the grouping was based on the transcript name and strand (-g 4,6) and computation was done on chr,start,end and transcript id (-c 1,2,3,4). The distinct count of transcript ids for verification

sort -k4,4 Ramb1LO2.bed | \

bedtools groupby -g 4,6 -c 1,2,3,4 -o distinct,min,max,count_distinct | \

awk '{OFS="\t";print $3,$4,$5,$1,$6,$2}' | \

sort -V -k1,2 > Ramb1LO2_groupby.bed

#Example output

CM028704.1 42238 46709 XM_027962292.1 1 -

CM028704.1 74992 78063 XR_003588699.1 1 -

CM028704.1 78522 79494 XR_003588700.1 1 +

CM028704.1 147143 150304 XM_027962305.1 1 -

CM028704.1 158201 167538 XM_027964169.1 1 +

CM028704.1 176125 178445 XM_027964177.1 1 -

CM028704.1 183267 193065 XM_027962318.1 1 -

#Checking the total number of records in the final sorted BED file.

wc -l Ramb1LO2_groupby.bed

49899 Ramb1LO2_groupby.bed

awk '$5!=1' Ramb1LO2_groupby.bed | wc -l

0

#Extracting exonic level FASTA sequences

bedtools getfasta \

-fi GCA_016772045.1_ARS-UI_Ramb_v2.0_genomic.fna \

-bed Ramb1LO2.bed \

-s -split -nameOnly > Ramb1LO2_NCBI103_geneBank_exons.fa

#Appending all exonic sequences from the same transcript id in the correct order

awk '/^>/ {if(prev!=$0) {prev=$0;printf("\n%s\n",$0);} next;} {printf("%s",$0);} END {printf("\n");}' \

Ramb1LO2_NCBI103_geneBank_exons.fa > Ramb1LO2_NCBI103_geneBank_rna.fa

#Cleaning up the strand information from the fasta header

sed -i 's/(-)//g;s/(+)//g' Ramb1LO2_NCBI103_geneBank_rna.fa

#Buidling Kallisto index for the quantification step.

samtools faidx Ramb1LO2_NCBI103_geneBank_rna.fa

kallisto index -i Ramb1LO2_NCBI103.idx Ramb1LO2_NCBI103_geneBank_rna.fa

################################## TPM expression estimation

#!/bin/bash

#SGE flags

#$ -l h_rt=4:00:00

#$ -l h_vmem=8G

#$ -pe sharedmem 4

#$ -V

#$ -t 1-61

#Required modules

module load pigz/2.3.3

module load kallisto/0.44.0

#Kallisto runs

kallisto quant --bias -t ${vcpu} -i Ramb2_refseq104.idx -o ${sra_id}_kallisto_Ramb2 <(zcat ${infile}) <(zcat ${infile/_1P.fq.gz/_2P.fq.gz})

kallisto quant --bias -t ${vcpu} -i Ramb1_NCBI103.idx -o ${sra_id}_kallisto_Ramb1 <(zcat ${infile}) <(zcat ${infile/_1P.fq.gz/_2P.fq.gz})

kallisto quant --bias -t ${vcpu} -i Ramb1LO2_NCBI103.idx -o ${sra_id}_kallisto_Ramb1LO2 <(zcat ${infile}) <(zcat ${infile/_1P.fq.gz/_2P.fq.gz})
