# Supplementary material for: An improved ovine reference genome assembly to facilitate in-depth functional annotation of the sheep genome
Source: Gigascience. 2022 Feb 4;11:giab096. doi: 10.1093/gigascience/giab096 (PMC8848310; doi:10.1093/gigascience/giab096)
Supplement: giab096_Response_to_Reviewer_Comments_Revision_1 [file giab096_response_to_reviewer_comments_revision_1.pdf]

Author's Response To Reviewer Comments

Close

Dear Hans,

Here is the manuscript updated to include the Oxford Nanopore sequence accession numbers (line 175), the updated BUSCO results (lines 321-328), and the GigaDB reference in the "Availability of supporting data" section (line 410).

Regards,
Ben

Close
