# Supplementary material for: An improved ovine reference genome assembly to facilitate in-depth functional annotation of the sheep genome
Source: Gigascience. 2022 Feb 4;11:giab096. doi: 10.1093/gigascience/giab096 (PMC8848310; doi:10.1093/gigascience/giab096)
Supplement: giab096_Reviewer_1_Report_Original_Submission — Aaron Shafer -- 8/30/2021 Reviewed [file giab096_reviewer_1_report_original_submission.pdf]

Reviewer Report

Title: An improved ovine reference genome assembly to facilitate in depth functional annotation of the sheep genome

Version: Original Submission **Date:** 8/30/2021

Reviewer name: Aaron Shafer

Reviewer Comments to Author:

My comments are minimal as the paper is succint. The authors present an improved genome, largely in the form of contiguity, and provide a number of statistics to support their argument. There are literally dozens upon dozens of different wayts to assemble and polish a genome and I see no value in suggesting changes in this regard as the approach more-or-less reflects the state-of-the-art. I also might question the description of "substantial improvement" as this really reflects the improvement in contiguity and less so the BUSCO, annotation. Also, scaffold L50 of the two available genomes is quite good, but not reported in Table 1, which I would suggest. The other Oar reference genomes were published 4 and 6 years ago, with this study offering the addition of nanopore sequence.
Minor
L55 - long read vs long sequence? Do you mean contigs or scaffolds?
L233- does freebayes do polishing? This is what is suggested by the current wording

Level of Interest

Please indicate how interesting you found the manuscript: Choose an item.

Quality of Written English

Please indicate the quality of language in the manuscript: Choose an item.

Declaration of Competing Interests

Please complete a declaration of competing interests, considering the following questions:

- Have you in the past five years received reimbursements, fees, funding, or salary from an organisation that may in any way gain or lose financially from the publication of this manuscript, either now or in the future?
- Do you hold any stocks or shares in an organisation that may in any way gain or lose financially from the publication of this manuscript, either now or in the future?
- Do you hold or are you currently applying for any patents relating to the content of the manuscript?

- Have you received reimbursements, fees, funding, or salary from an organization that holds or has applied for patents relating to the content of the manuscript?
- Do you have any other financial competing interests?
- Do you have any non-financial competing interests in relation to this paper?

If you can answer no to all of the above, write 'I declare that I have no competing interests' below. If your reply is yes to any, please give details below.

I declare that I have no competing interests

I agree to the open peer review policy of the journal. I understand that my name will be included on my report to the authors and, if the manuscript is accepted for publication, my named report including any attachments I upload will be posted on the website along with the authors' responses. I agree for my report to be made available under an Open Access Creative Commons CC-BY license (http://creativecommons.org/licenses/by/4.0/). I understand that any comments which I do not wish to be included in my named report can be included as confidential comments to the editors, which will not be published.

Choose an item.

To further support our reviewers, we have joined with Publons, where you can gain additional credit to further highlight your hard work (see: https://publons.com/journal/530/gigascience). On publication of this paper, your review will be automatically added to Publons, you can then choose whether or not to claim your Publons credit. I understand this statement.

Yes Choose an item.
